# Unravelling the Electro‐Photocatalytic Water Splitting Capabilities of 2D‐Bifunctional Mo_2_S_3_‐WS_2_ Catalyst: Implications for Renewable Energy Platforms

**DOI:** 10.1002/smtd.202500476

**Published:** 2025-07-26

**Authors:** Levna Chacko, Amutha Subramani, Jakub Regner, Pradip Kumar Roy, Rui Gusmão, Roussin Lontio Fomekong, Shuangying Wei, Vlastimil Mazánek, Zdeněk Sofer

**Affiliations:** ^1^ Department of Inorganic Chemistry University of Chemistry and Technology Prague Technicka 5 Prague 6 16628 Czech Republic; ^2^ Centre of Excellence ENSEMBLE3 Sp. z o. o Wolczynska Str. 133 Warsaw 01‐919 Poland; ^3^ Higher Teacher Training College University of Yaounde I P.O. BOX 47 Yaounde Cameroon

**Keywords:** 2D materials, molybdenum chalcogenide, photocatalysis, water splitting

## Abstract

Addressing the energy crisis and environmental pollution demands the development of efficient electro‐ and photocatalysts capable of facilitating hydrogen production via water splitting. Engineering semiconductor‐based 2D heterostructures with remarkable visible‐light harvesting capability proves to be an effective approach for constructing profoundly active electro‐photocatalytic entities. This paper focuses on designing a heterostructure comprising tungsten disulphide (WS_2_) integrated with molybdenum sesquisulfide (Mo_2_S_3_) to increase electrocatalytic activity. Moreover, nanoengineering the 2D/2D heterostructure with varying lateral dimensions increases the density of catalytic active sites. Due to its superior geometric configuration, the polarization curves of the Mo_2_S_3_‐WS_2_ composite display outstanding characteristics as a bifunctional electro‐photocatalyst, with low overpotentials (η) of 92 mV for hydrogen evolution reaction (HER) and 310 mV for oxygen evolution reaction (OER) at a current density of 10 mA cm^2^, maintaining stability for up to 100 h. The electrolyzer achieved a current density of 1 A cm^2^ with a cell voltage of 2.07 V, corresponding to a voltage efficiency of 72%. Integrating the electrolyzer with a commercial silicon solar cell enabled efficient solar‐driven water splitting, reaching a current density of ≈0.8 A cm^2^ at an applied voltage of 2 V. This work provides valuable insights into the design of innovative hetero‐nanostructures with superior catalytic properties for water splitting, contributing to a sustainable energy future.

## Graphical abstract

Schematic representation of band alignment and scanning transmission electron microscopy (STEM) image of Mo_2_S_3_‐WS_2_ nanocomposites.

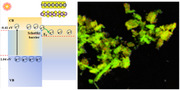

## 1 Introduction

To address the worldwide energy demand and the concerns of energy scarcity due to the depletion of fossil fuels, the scientific community has been working hard to develop new energy options to meet the demand for sustainable energy while environmental protection. Consequently, deriving clean energy using renewable energy sources is identified to be an efficient technology for achieving carbon neutrality. In that aspect, the clean and abundant solar energy has received considerable research interest and popularity in recent years. With high gravimetric energy density of 142 MJ mol^−1^ compared to commonly used fuels like gasoline (≈46 MJ kg^−1^), natural gas (≈44 MJ kg^−1^) along with zero‐carbon and zero‐pollutant emission, hydrogen produced from renewable solar energy has been envisaged as an alternative clean, environmentally friendly energy carrier that can reduce the dependency on hydrocarbon‐based fuels leading to decarbonization and defossilization. Being a green and sustainable method to produce hydrogen, photocatalytic water splitting for H_2_ generation has emerged as an efficient technique for mass production of high‐purity H_2_. With two half‐cell reactions: the HER and the sluggish OER, water splitting must overcome an internal energy barrier of 237.2 kJ mol^−1^. As a result, it is imperative to design efficient, earth abundant, cost‐effective catalysts with high redox kinetics and enhanced stability. Furthermore, significant challenges such as narrow light absorption, fast recombination, slow redox kinetics, poor charge separation and transport hinder the achievement of efficient photocatalytic hydrogen generation. In this regard, developing innovative semiconductor photocatalysts with high solar‐to‐hydrogen conversion efficiency has risen to the top of the research agenda. The efficient dissociation of water molecules leading to H_2_ and O_2_ generation by TiO_2_ nanostructures via solar‐driven water splitting, as demonstrated by Fujishima and Honda,^[^ ^1^ ^]^ marked the beginning of light‐assisted clean energy production.

Research investigations have explored several non‐noble catalysts for hydrogen production, including metal chalcogenides (MoS_2_, WS_2_, CdS),^[^ ^2^, ^3^, ^4^ ^]^ metal phosphides (NiP, Cu_3_P, CoP, WP),^[^ ^5^, ^6^, ^7^ ^]^ oxides (ZnO, NiO, TiO_2_, SnO_2_),^[^ ^8^, ^9^ ^]^ doped, functionalized and composite heterostructures.^[^ ^10^, ^11^, ^12^, ^13^ ^]^ Among various photocatalysts, nanocomposite materials with hetero‐interfaces have garnered significant research interest due to their distinctive electronic and geometric structures, which facilitate efficient light harvesting, carrier charge separation and transport, and redox kinetics resulting in excellent catalytic activity. 2D materials with excellent conductivity, high specific surface area, atomic thickness, excellent charge transport properties, tunable electronic structure, and band gap, present themselves as superior options for hydrogen evolution catalysts. As members of transition metal dichalcogenide (TMDC) family, molybdenum‐sulfur (Mo‐S) and tungsten‐sulfur (W‐S) based materials stand out as promising materials, exhibiting an indirect‐to‐direct band gap transition with a reduction in the number of layers. Additionally, considering the Gibb's free energy (ΔG_H_) for hydrogen adsorption, a key descriptor for hydrogen evolution, Mo, W and S edge sites have been found to exhibit near‐thermoneutral hydrogen binding energy.^[^ ^14^, ^15^ ^]^ WS_2_ consists of three atomic planes of S‐W‐S atoms with a strong in‐plane covalent bonding and out‐of‐plane Van der Waals intermolecular interactions. It is non‐toxic, inexpensive, exhibits excellent conductivity and a high specific area with abundant active sites, and more importantly, possesses a narrow band gap making it more prone to photoexcitation for generating active species. Fulfilling the preferred conditions required for photocatalytic water splitting, WS_2_ possesses an indirect band gap of 1.2 eV and a direct band gap of ≈2.1 eV^[^ ^16^ ^]^ in the visible light range with broad absorption in the visible spectrum ranging from 350 – 700 nm.^[^ ^17^ ^]^

Besides, the conduction band and valence band potentials of WS_2_ is −3.61 and −5.98 eV, relative to the oxidation and reduction potentials of H_2_O, create thermodynamically favorable conditions for water splitting, where the $E_{H^{+}/H_{2}}$ and $E_{O_{2}/H_{2}O}$ values relative to the vacuum level endure to be −4.44 and −5.67 eV, respectively.^[^ ^18^ ^]^ Nevertheless, employing WS_2_ as a sole catalyst in water‐splitting systems has encountered numerous challenges, including rapid recombination of generated electrons and holes, as well as low efficiency in charge transfer.

However, Despite the promising attributes of various photocatalysts, including WS₂, several critical limitations persist. Materials such as TiO_2_, MoS_2_, and other TMDCs have been extensively studied; however, they frequently exhibit narrow light absorption spectra, low charge carrier mobility, and rapid recombination of photogenerated electron–hole pairs. Specifically, WS_2_ alone demonstrates significant recombination losses and limited interlayer charge transport, which impede its photocatalytic efficiency.

Therefore, it is crucial to enhance the photocatalytic activity of semiconducting WS_2_ by creating an active composite photocatalyst, thereby tuning the electronic structure, improving the absorption of visible light and significantly slowing down the recombination of photo‐generated electron‐hole pairs. Incorporating a Mo‐S compound, Mo_2_S_3_ with metallic characteristics and excellent conductivity is expected to reduce the transfer resistance of photo‐induced electrons at the Mo_2_S_3_/WS_2_ interface. Unlike semiconducting MoS_2_, Mo_2_S_3_ manifest a quasi‐layered crystal structure with Mo atoms no longer serving as the centers of sulfur octahedra. Rather, it consists of two distinct zig‐zag chains of Mo‐Mo bonding along the monoclinic b‐axis contributing toward exceptional transport properties. In general, Mo_2_S_3_ can essentially be visualized as molecular Mo‐S chains intercalated within the 1T’‐MoS_2_ structure.^[^ ^19^ ^]^ Zhang et al. have reported notable photocatalytic activity in Mo_2_S_3_/BiOBr,^[^ ^20^ ^]^ ascribed to the fast transfer of photogenerated carriers between materials ensuring efficient separation of photogenerated charges. Further reports have also demonstrated that the heterojunction of Mo_2_S_3_ with Co_3_S_4_,^[^ ^21^ ^]^ Bi_2_O_2_CO_3_^[^ ^22^ ^]^ offer more efficient pathways for electron transport, enhancing redox processes.

Considering these factors, this work pioneers the development of Mo_2_S_3_‐WS_2_ based heterostructures for electro‐photocatalytic hydrogen production. The active heterostructure with a maximized synergistic interaction and effective exposure of surface‐active sites is found to improve the energy conversion. The systematically designed heterostructure, featuring a 2D structure with varying lateral dimensions, displays outstanding electro‐photocatalytic performance. It displays a low η of 145 mV for electro‐catalytic HER and 92 mV for photo‐catalytic HER and 420 mV for electro and 310 mV for photo‐catalytic OER at a current density of 10 mA cm^2^. When incorporated as a bifunctional two‐electrode system enabled water electrolysis at ≈2.16 V at a current density of 100 mA cm^2^. A comprehensive theoretical approach was also adopted to substantiate the potential electro‐photocatalytic activity of Mo_2_S_3_‐WS_2_ for overall water splitting. Additionally, when integrated into a Mo_2_S_3_‐WS_2_ || Mo_2_S_3_‐WS_2_ zero‐gap alkaline water electrolyzer (AWE), it shows low cell voltages of 2.07 V at room temperature and 1.95 V at 60 °C, maintaining excellent stability for a long duration of operation at high current density. Furthermore, coupling the electrolyzer with a commercial silicon solar cell allowed for an evaluation of solar‐driven water‐splitting performance.

## 2 Results and Discussion

**Figure 1:**
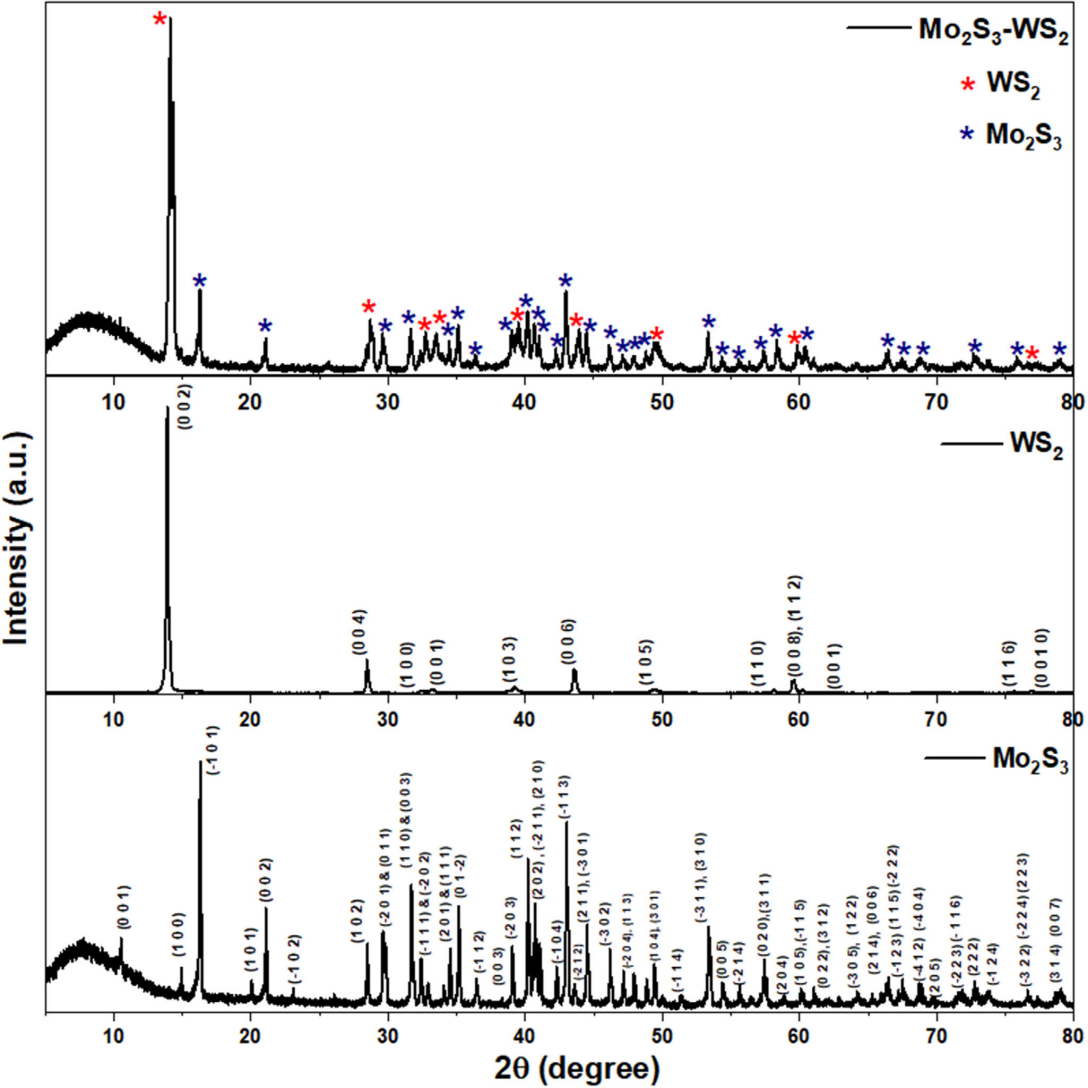
XRD of Mo_2_S_3_, WS_2_ and Mo_2_S_3_‐WS_2_ nanostructures.

X‐ray diffraction (XRD) measurements were conducted to analyze the crystal structures and phase purity of the as‐synthesized samples, as depicted in **Figure** **1**. Unlike 2H‐MoS_2_, Mo_2_S_3_ possesses a fundamental structure, wherein the Mo atoms are displaced from their ideal positions, constituting two crystallographically independent types of zig‐zag chains of Mo atoms along the monoclinic b‐axis direction where the Mo atoms occupy 2/3 of the octahedral interstices. The plane of type 1 zig‐zag chain is almost parallel to (‐1 0 1), while the type 2 zig‐zag chain to (0 0 1) plane.^[^ ^23^ ^]^ As evident from Figure 1 and Figure S2 (Supporting Information), the XRD patterns for the Mo_2_S_3_ samples represent the formation of monoclinic, highly crystalline Mo_2_S_3_ nanostructures with space group P21/m, exhibiting high purity and quality (Ref. Code: 04‐003‐4491). Mo_2_S_3_ appears to have a preferential orientation along the b axis, as evidenced by the presence and significant increase in intensity seen in the diffraction peaks along (‐1 0 1) and (0 0 1) planes. The XRD pattern corroborates the Mo‐S phase diagram, affirming the formation and stabilization of the Mo_2_S_3_ structure at temperatures exceeding 950 K.^[^ ^24^ ^]^ Similarly, the XRD patterns also confirmed the formation of hexagonal WS_2_ nanostructures (Figure 1; Figure S3, Supporting Information) with the space group P63/mmc (Ref. Code: 04‐003‐5636). The successful formation of the Mo_2_S_3_‐WS_2_ composite structures was also confirmed by the XRD pattern, which displays peaks corresponding to both Mo_2_S_3_ and WS_2_.

**Figure 2:**
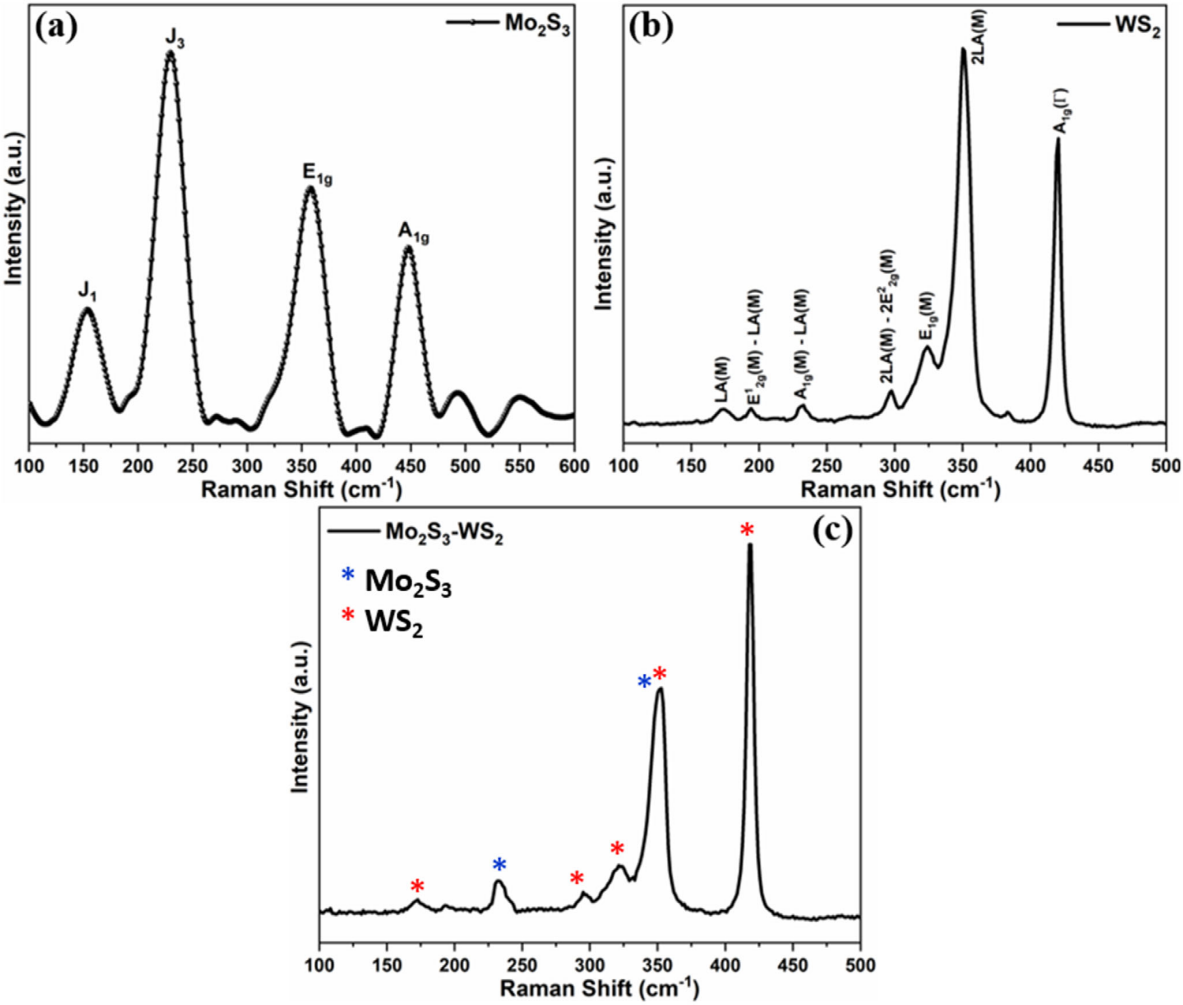
Raman spectroscopic images of a) Mo_2_S_3_, b) WS_2_ and c) Mo_2_S_3_‐WS_2_ nanostructures.

Further examination of the structural and electronic characteristics of the Mo_2_S_3_, WS_2_ and Mo_2_S_3_‐WS_2_ nanostructures was performed using Raman spectroscopy, a powerful method that examines the inelastic scattering of light to investigate the vibrational modes of materials as depicted in **Figure** **2**. Raman bands within the range of 100 to 500 cm^−1^ correspond to the characteristic vibration modes of Mo_2_S_3_ (Figure 2a), with the recorded peaks ≈150, 237 and 450 cm^−1^ corresponding to the J_1_, J_2_ and A_1g_ vibrational modes respectively for 1T’‐MoS_2_ with the Mo_2_S_3_ structure.^[^ ^25^ ^]^ Additionally, the distinctive Raman signal observed at 350 cm^−1^ is attributed to the octahedral coordination of Mo atoms and this vibrational mode is found to be absent in 2H‐MoS_2_.^[^ ^26^ ^]^ The E_1g_ mode represents the in‐plane vibration, while the A_1g_ mode is associated with the out‐of‐plane vibrations involving molybdenum and sulfur atoms. The Raman spectrum of WS_2_ exhibits a notably enriched profile similar to the Raman scattering spectra observed in previous reports upon excitation with 532 nm corresponding to various vibrational modes.^[^ ^27^ ^]^ As observed in Figure 2b, the Raman spectra display numerous second‐order peaks compared to those observed for the bulk material with the 2LA(M) and A_1g_(Γ) phonon modes being the strongest Raman peaks.^[^ ^28^ ^]^ When the energy of the incident laser light aligns with that of the A or B excitons in WS_2_, there is a notable increase in the Raman scattering intensities observed for these vibrational modes. The A_1g_(Γ) peak is indicative of the out‐of the‐plane vibration of sulfur atoms relative to the tungsten atom at the Γ point of the Brillouin zone. Sulfur atoms in this mode of vibration migrate perpendicular to the plane of the WS_2_ layer.

In contrast, the 2LA(M) mode is indicative of the second‐order Raman scattering process, which involves the interaction of two longitudinal acoustic (LA) phonons located at the M point within the Brillouin zone. LA phonons encompass the atomic vibrations aligned parallel to the direction of wave propagation. Furthermore, the 2LA(M) and A1g(Γ) phonon modes of WS_2_ provide intriguing insights into how dimensionality affects the vibrational behavior of the material. One such notable phenomenon observed is the contrasting trends in the intensity of these vibrational modes, indicative of the dimensionality or number of layers. The absolute intensity of the longitudinal acoustic phonon, 2LA(M), demonstrates an increase as the number of layers decreases. The enhanced intensity is indicative of the stronger confinement effects and increased phonon coherence length, resulting in more prominent intra‐layer electronic interactions within the layers with a decrease in the number of layers. On the contrary, the intensity of the A_1g_(Γ) mode exhibits a decrease with decreasing number of layers. A_1g_(Γ) mode being predominantly sensitive to interlayer interactions, the reduced phonon restoring forces acting on the vibrating sulfur atoms resulting from weaker interlayer coupling in thinner WS_2_ layers is found to significantly lessen the intensity of the Raman scattering.^[^ ^29^ ^]^ The Raman spectrum of WS_2_ is also characterized by several prominent peaks located at ≈174, 194, 232, 297, 350 and 420 cm^−1^, each associated with specific vibrational modes.^[^ ^30^, ^31^, ^32^ ^]^ The peak at 174 cm^−1^ is a second‐order defect‐activated longitudinal acoustic phonon mode near the M point of the Brillouin zone, involving the vibration of atoms along the same direction as the wave propagation. The Raman mode at ≈194 cm^−1^ corresponds to the energy difference between the E^1^_2g_ phonon mode and the longitudinal acoustic phonon mode, showcasing the in‐plane vibration of sulfur atoms in opposite directions. A_1g_(M) – LA(M) vibrational mode at ≈232 cm^−1^ also originates the interaction between two Raman‐active phonons, the A_1g_ and the LA phonon mode, further reflecting the out‐of‐plane vibration of sulfur and tungsten atoms in opposite directions. The difference mode 2LA(M) – 2E^2^_2g_(M) at ≈297 cm^−1^ originates from the combination of two LA and two E^2^_2g_ phonon modes, where E_2g_ indicates the in‐plane vibrational motion in the plane of sulfur atoms along the direction of the wave vector M. The E_1g_(M) mode observed at 324 cm^−1^ represents the vibration corresponding to the E_1g_ phonon modes at the M point of the Brillouin zone. Finally, the A_1g_ Raman mode ≈420 cm^−1^ represents the out‐of‐plane vibrations of sulfur atoms. The Raman spectra of the Mo_2_S_3_‐WS_2_ composites (Figure 2c) displayed the distinctive peaks of WS_2_ and Mo_2_S_3_ without any additional peaks, implying that the composites consisted solely of these two components and that these components maintain their individual crystal structures within the composite.

**Figure 3:**
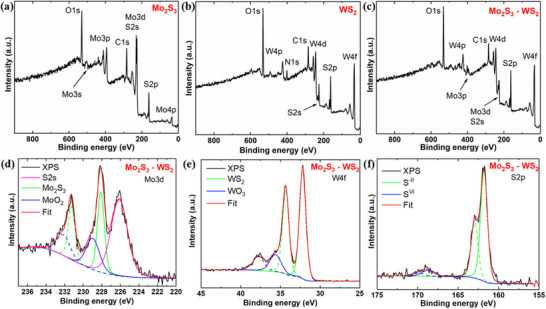
XPS survey spectra of a) Mo_2_S_3_, b) WS_2_, c) Mo_2_S_3_‐WS_2_ nanostructures and XPS high‐resolution spectra d) Mo 3d, e) W 4f and f) S 2p of Mo_2_S_3_‐WS_2_ nanostructures.

The surface chemical state and the elemental composition of the materials were effectively characterized with X‐ray photoelectron spectroscopy (XPS), a potent technique for examining the surface chemistry of materials. The survey XPS spectra shown in **Figure** **3**a–c attest to the existence of Mo, S, and W in their corresponding compounds as well as their coexistence in the composite material. In the XPS findings of Mo_2_S_3_ (Figure S4a, Supporting Information), the characteristic Mo peaks at 231.3 eV (Mo 3d_3/2_) and 227.9 eV (Mo 3d_5/2_) confirm the formation of Mo^3+^ state together with a small peak at 225.8 eV originating from S 2s orbital. These Mo peaks observed in Mo_2_S_3_ is found to be red‐shifted by ≈1.2 eV compared with the Mo^4+^ peaks with binding energies of 232.3 (Mo 3d_3/2_) and 229.2 eV (Mo 3d_5/2_) observed in MoS_2_.^[^ ^33^, ^34^ ^]^ XPS spectra confirm that the Mo atoms are indeed in the +3 oxidation state within Mo_2_S_3_, further supporting the formation of pure Mo_2_S_3_ structures. The S2p XPS spectra of Mo_2_S_3_ (Figure S4b, Supporting Information), related to S 2p_3/2_ and 2p_1/2_ were centered at binding energy of 161.9 and 162.8 eV representing S^2‐^ state. Additionally, the peak observed at 168.6 eV suggests the existence of oxidized sulfur contents.^[^ ^35^ ^]^ The high‐resolution XPS spectra of WS_2_ (Figure S4c, Supporting Information) displayed binding energy at 32.5, 34.5, and 38.1 corresponding to W 4f_7/2_, W 4f_5/2_ and W 5p_3/2_.^[^ ^36^ ^]^ The binding energies of S 2p_3/2_ and S 2p_1/2_ were identified in the WS_2_ S2p spectra at 160.8 and 161.9 eV, respectively (Figure S4d, Supporting Information), confirming metal sulfur bonds. Similarly, the presence of peak ≈167.8 eV suggests the presence of sulfur atoms in a higher oxidation state, especially S^6+^ state.^[^ ^37^ ^]^ Compared to pure Mo_2_S_3_ and WS_2_, the binding energies of the elements Mo, W and S in Mo_2_S_3_‐WS_2_ composites exhibited slight shifts (Figure 3d–f). These shifts suggest the potential occurrence of interactions between Mo_2_S_3_ and WS_2_ within the composite material.

**Figure 4:**
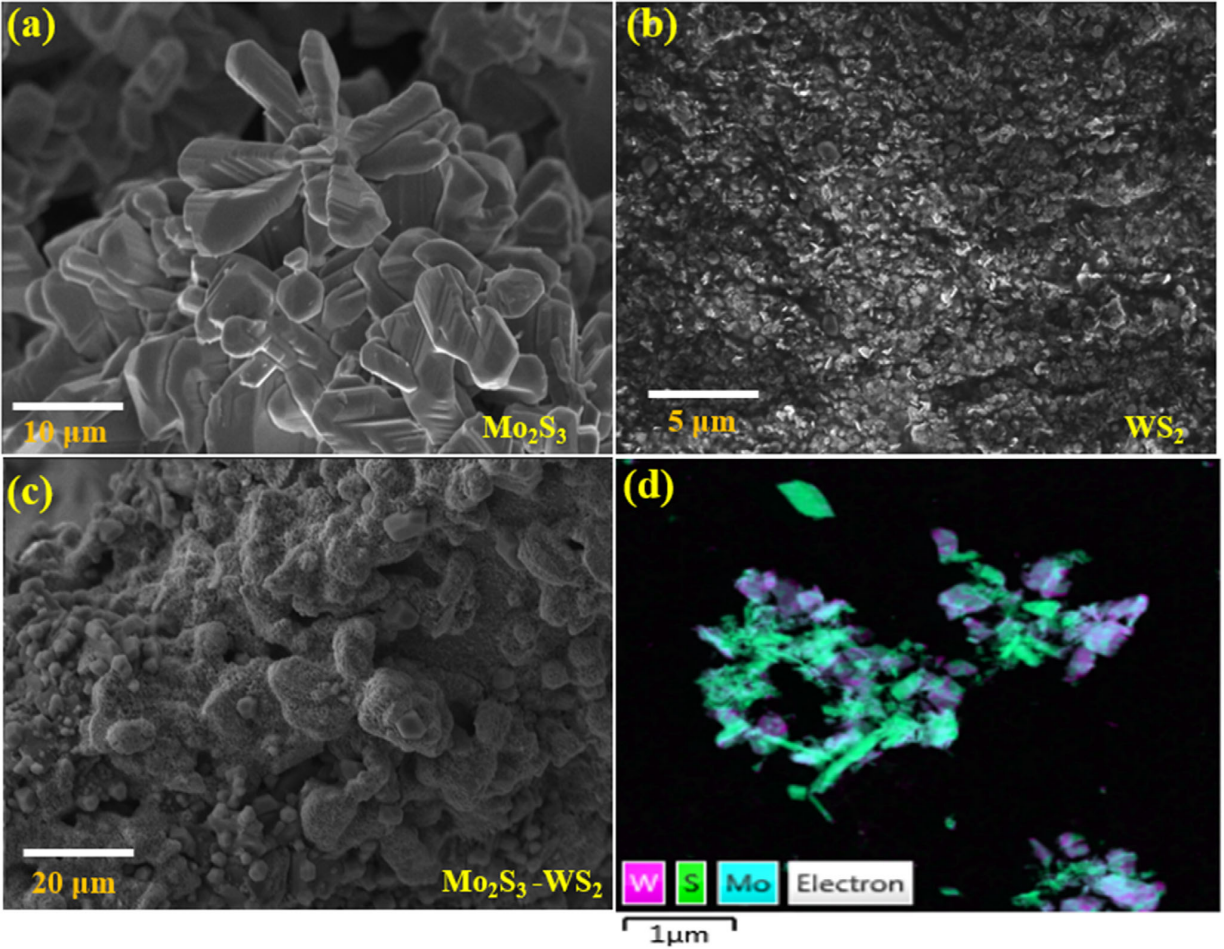
Scanning electron microscope images of a) Mo_2_S_3_, b) WS_2_, c) Mo_2_S_3_‐WS_2_ and d) STEM‐EDX mapping of Mo_2_S_3_‐WS_2_.

**Figure 5:**
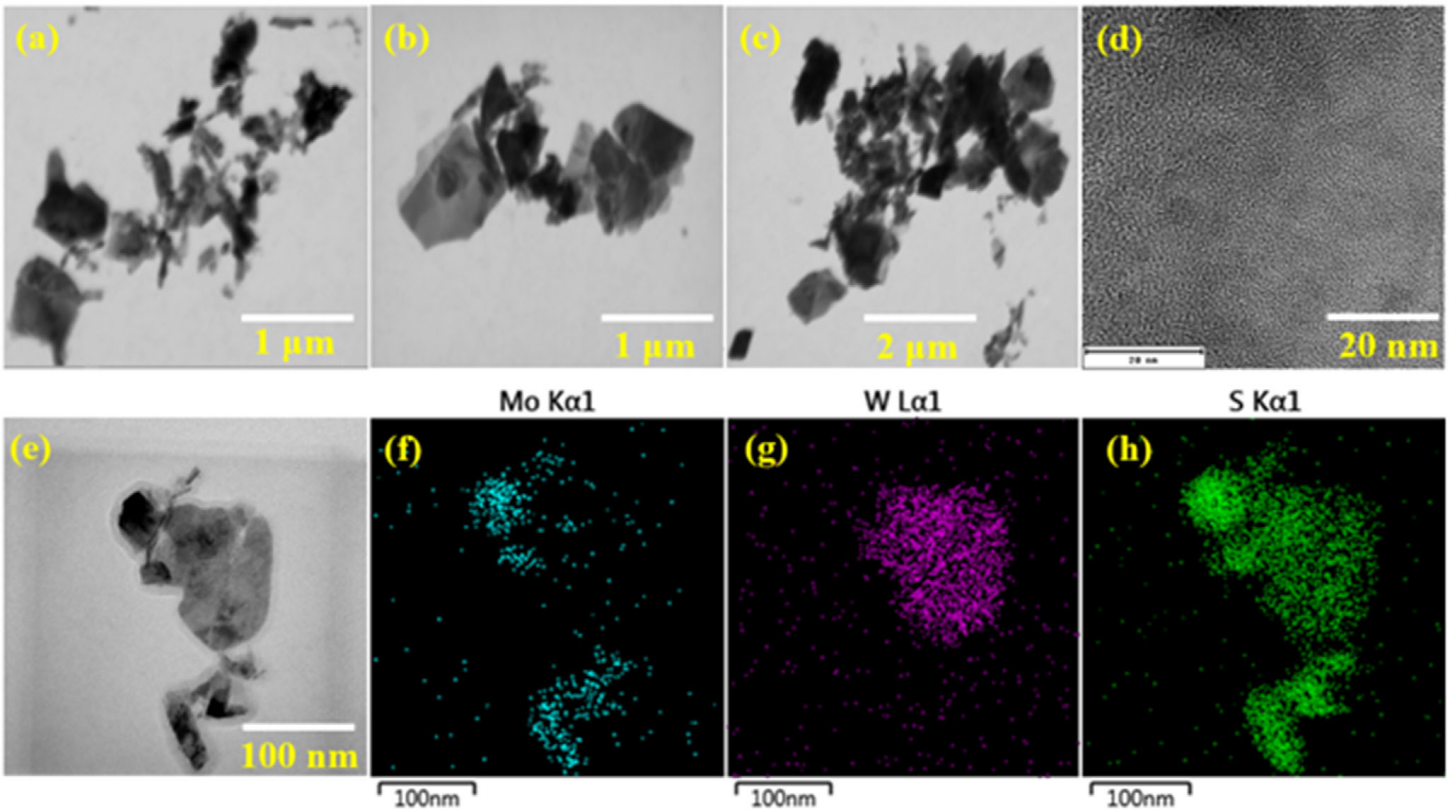
a–c) STEM images, d–h) TEM image and corresponding EDX elemental mapping.

The morphological features of Mo_2_S_3_, WS_2_ and Mo_2_S_3_ nanohybrids incorporating WS_2_ were examined using scanning electron microscopy (SEM) together with energy dispersive X‐ray spectrometry (EDX) mapping to analyze the elemental distribution. As shown in **Figure** **4**a, Mo_2_S_3_ nanostructures possess a quasi‐two‐dimensional structure with almost uniform distribution, while WS_2_ is found to showcase 2D nanostructures with very small and uniform lateral dimensions (Figure 4b) compared to Mo_2_S_3_. The dispersion of WS_2_ in Mo_2_S_3_ resulted in the formation of a unique nanohybrid structure (Figure 4c), ascribed to the self‐assembly of WS_2_ on quasi‐2D Mo_2_S_3_ during the sonication process. Quantitative EDX analysis confirms the presence of Mo_2_S_3_ and WS_2_ with an elemental ratio of Mo:S equal to 2:3 (Figure S5, Supporting Information) and that of W:S equal to 1:2 (Figure S6, Supporting Information). Similarly, the STEM and STEM – EDX mapping of the Mo_2_S_3_‐WS_2_ composite also indicate the uniform distribution and presence of Mo, W, and S within the resultant nanohybrids as represented in Figure 4d and **Figure** **5**a–c, Figures S7 and S8 (Supporting Information) respectively. Transmission electron microscope (TEM), high‐resolution TEM (HRTEM) and elemental analysis was performed to further confirm the formation of the heterostructured Mo_2_S_3_‐WS_2_ composite.

The HR TEM characterization further confirmed the formation of the Mo_2_S_3_‐WS_2_ heterostructure through crystallographic analysis, as shown in Figure S9 (Supporting Information). The EDX spectra on two located spots show a prevalence of each component of the composite in different zones of the observed view field. HR‐TEM analysis revealed crystallographically distinct lattice periodicities with spatial resolution, displaying well‐defined fringe patterns indicative of high crystallinity and minimal defect density at the heterojunction.^[^ ^38^ ^]^ Systematic fast Fourier transform analysis allowed for the estimation of interplanar d‐spacings of 0.3517 nm corresponding to the Mo_2_S_3_ (010) crystallographic plane within orthorhombic *Pnma* space group symmetry, and 0.3309 nm indexing to the 2H‐WS_2_ (100) plane. The HR micrographs substantiate morphologically distinct Mo_2_S_3_ and WS_2_ nanosheets exhibiting close surface adhesion, likely through the van der Waals epitaxial registry. These crystallographically interfacial interactions optimize the efficiency of charge separation across heterostructured domains. The cross‐matching of EDX mapping of Mo, W and S elements, and micrograph analysis reveals systematic crystallographic facet organization with (010) facets of orthorhombic Mo_2_S_3_ visible on the left side of the demarcation line, as shown in Figure S9 (Supporting Information), while lighter contrast regions represent hexagonal WS_2_ domains. The delineation of the interface mapping estimation confirms heterostructure formation with preserved crystallographic integrity for the sub‐components transition metal‐sulfur phases, establishing optimized electronic band alignment for enhanced photocatalytic functionality through systematic defect engineering protocols.

**Figure 6:**
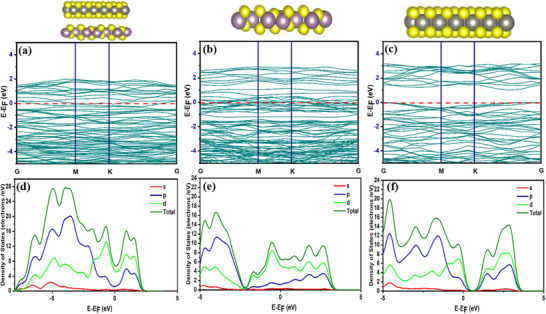
Electronic structure of a) Mo_2_S_3_‐WS_2_, b) Mo_2_S_3_, c) WS_2_ and partial and total density of states of d) Mo_2_S_3_‐WS_2_, e) Mo_2_S_3_ and f) WS_2_.

To gain a better understanding of the structural and electronic properties of the Mo_2_S_3_‐WS_2_ heterostructure, we first built models of the Mo_2_S_3_ and WS_2_ monolayers, followed by the Mo_2_S_3_‐WS_2_ heterostructure (**Figure** **6**a). The band gap for Mo_2_S_3_ and WS_2_ monolayers is 0^[^ ^39^ ^]^ and 1.45 eV,^[^ ^40^ ^]^ respectively, which is consistent with the theoretical data shown in Figure 6b,c respectively. Furthermore, the Mo_2_S_3_‐WS_2_ heterostructure shows a shift of the conduction band close to the Fermi level, resulting in metallic behavior and band gap tuning at the gamma point, influencing the energy levels between the valence band (VB) and conduction band (CB) in the electronic structure as shown in Figure 6a. Some novel states appearing near the Fermi level because of the formation of Schottky junction, which introduces new electronic states and results in a modified charge distribution within the system. It can be concluded that the heterostructure of Mo_2_S_3_‐WS_2_ may promote electron transfer between the valence band and the conduction band of the material under light irradiation, which is consistent with the experimental results. The valence and conduction band electron contribution can be well understood with the density of states calculations. The total density of states (TDOS) and partial density of states (PDOS) for the Mo_2_S_3_‐WS_2_, Mo_2_S_3_ and WS_2_ are shown in Figure 6d–f. The overlap of W‐5d4 with S‐ 3p4 orbital below the Fermi level in the Figure 6d verifies the electronic states of WS_2_ in the Mo_2_S_3_‐WS_2_ heterostructure. Near the Fermi level, a strong 4d orbital contribution from Mo leads to the shift of conduction bands below the Fermi level, creating metallic behavior. This proves that the Mo_2_S_3_‐WS_2_ heterostructure has the greater influence on electronic distribution and improves conductivity. Additionally, the nature of bonding at the Mo_2_S_3_–WS_2_ interface exhibits a mixed character, combining covalent bonding with a partial ionic character due to charge polarization. Evidence of covalent interaction is provided by the projected density of states (PDOS), where the significant overlap between W‐5d and S‐3p orbitals below the Fermi level indicates orbital hybridization and electron sharing across the interface. Meanwhile, the dominant contribution of Mo‐4d near the Fermi level leads to a shift in the conduction bands and indicates a redistribution of the electronic charge. This redistribution, driven by differences in electronegativity between the Mo, W, and S atoms, introduces polarization within the covalent bonds, effectively giving them partial ionic character. Such polarization enhances internal electric fields and facilitates more efficient charge transport. Collectively, the Mo_2_S_3_–WS_2_ heterostructure is stabilized by strongly hybridized covalent bonds that are partially polarized, directly contributing directly to the improved conductivity and enhanced catalytic activity observed in HER and OER.

**Figure 7:**
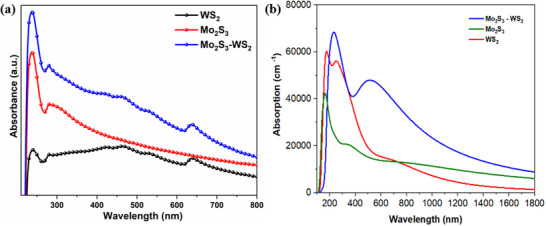
UV–vis absorbance spectra of Mo_2_S_3_, WS_2_ and Mo_2_S_3_‐WS_2_ composite, a) experimental and b) theoretical data.

Understanding the UV–vis absorption spectra of nanostructures is essential for gaining insights into their electronic structures and excitonic behaviors, which are critical for many applications. The performance of a photocatalyst, in particular, depends significantly on its ability to absorb and harness light energy efficiently. **Figure** **7**a showcases the UV–vis absorbance spectra obtained experimentally for WS_2_, Mo_2_S_3_ and the Mo_2_S_3_‐WS_2_ composite, offering a comprehensive examination of their optical properties. Compared to the bulk structure, WS_2_ nanostructures exhibit unique optical properties when their size is reduced to the nanoscale. Due to their semi‐conducting nature, they exhibit a rich absorption spectrum with distinct peaks visible at the nanoscale, arising from different electronic transitions and indicating the presence of significant energy states within the material.^[^ ^41^, ^42^ ^]^ The A and B excitonic peaks observed ≈638 and 530 nm correspond to direct gap excitonic transitions at the K‐point in the Brillouin zone and are the lowest energy exciton states. These peaks emanate from spin‐orbit coupling‐induced valence band splitting and there exists an energy separation of ≈0.4 eV between these peaks. The peaks observed around 464 and 420 nm are attributed to the high‐energy excitons, characterizing the transitions between the density‐of‐states in the valence and conduction bands between the Λ and Г space in the Brillouin zone.^[^ ^43^ ^]^ The optical signature of the WS_2_ nanostructures reveals the interaction between the excitonic attributes and the few‐layered morphology of the WS_2_, further confirming quantum confinement and strong Coulombic interactions. For Mo_2_S_3_, which exhibits a metallic nature, it lacks excitonic absorption and instead displays a broad absorbance spectrum. A small peak observed ≈284 nm confirms the photoexcitation of electrons from the valence band to the conduction band.^[^ ^44^ ^]^ Furthermore, the absorption spectrum of the Mo_2_S_3_‐WS_2_ composite material indicates broadened light absorption and distinct excitonic peaks that confirm the formation of the Mo_2_S_3_‐WS_2_ structure. In relation to the experimental UV–vis absorbance spectra (Figure 7a), the optical properties of the nanostructures contributing to photocatalytic activity were theoretically calculated (Figure 7b). The figure reveals the visible light response of the Mo_2_S_3_‐WS_2_ structure showing an absorption edge near 380 nm.

**Figure 8:**
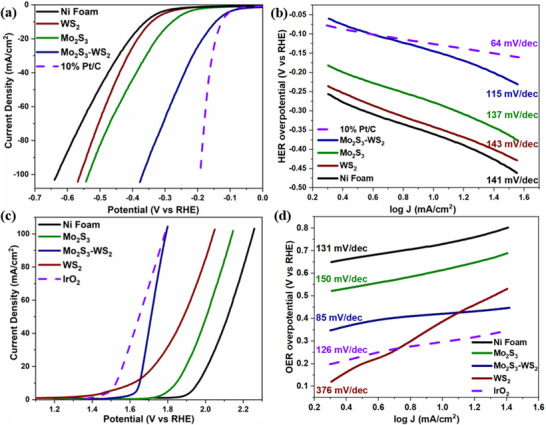
a) Linear sweep voltammetry (LSV) curves at 5 mV s^−1^ sweep rate representing HER, b) Tafel slope, a) Linear sweep voltammetry (LSV) curves at 5 mV s^−1^ sweep rate representing OER and b) Tafel slope of Mo_2_S_3_, WS_2_ and Mo_2_S_3_‐WS_2_ catalysts in 1 M KOH solution.

Despite the development of numerous electrocatalysts for effective performance in cathodic HER or anodic OER, achieving catalysts that efficiently perform both half‐reactions in alkaline water splitting continues to pose a significant challenge. Here, the bifunctional catalytic performance of the Mo_2_S_3_‐WS_2_ composite was examined for HER and OER in a 1 M KOH solution using a three‐electrode setup. Commercial benchmark catalysts, 10 wt.% Pt/C for HER and IrO_2_ for OER were used for comparison. **Figure** **8**a depicts the linear sweep voltammetry (LSV) curve in the cathodic region, where the Mo_2_S_3_‐WS_2_ heterostructured catalyst demonstrates superior catalytic activity compared to pure Mo_2_S_3_ and WS_2_, with a low η of 145 mV at 10 mA cm^2^. The η for 10 wt.% Pt/C, pure Mo_2_S_3_, WS_2_, and Ni‐foam were 142, 279, 343, and 362 mV respectively. For electrochemical kinetics, Figure 8b reveals that the Tafel slope (TS) of Mo_2_S_3_‐WS_2_ is 115 mV dec^−1^, confirming the Volmer–Heyrovský steps act as the rate‐determining step (RDS) in the HER mechanism. Meanwhile, the TS for Mo_2_S_3_ and WS_2_ are 137 and 143 mV dec^−1^ respectively. This result confirms that the Volmer‐Heyrovský steps act as the RDS in the HER mechanism, which includes the electrochemical adsorption of hydrogen atoms on the catalyst surface followed by an electrochemical desorption process releasing hydrogen molecules.

Similarly, the OER activity of the catalysts was evaluated in a 1 M KOH solution, revealing that the Mo_2_S_3_‐WS_2_ catalyst exhibited a lower η and TS compared to the pure nanostructures (Figure 8c,d). The LSV curves revealed an η of 420 mV for the Mo_2_S_3_‐WS_2_ electrode at a current density of 10 mA cm^2^, compared to 300 mV for IrO_2_, 610 mV for Mo_2_S_3_, 380 mV for WS_2_ and 730 mV for Ni foam. Even at a higher current density of 100 mA cm^2^, the Mo_2_S_3_‐WS_2_ electrode demonstrates a low OER η of 560 mV, outperforming both Mo_2_S_3_ and WS_2_. Furthermore, the OER catalytic kinetics derived from Tafel plots highlight the favorable kinetics of the Mo_2_S_3_‐WS_2_ catalyst, which exhibits a low TS of 85 mV/dec, compared to Mo_2_S_3_ (150 mV dec^−1^) and WS_2_ (376 mV dec^−1^). The relatively low Tafel slope suggests that the RDS for Mo_2_S_3_‐WS_2_ likely involves a post‐electron transfer chemical step, such as O–O bond formation, following a rapid pre‐equilibrium electron transfer. In contrast, the higher Tafel slope observed for Mo_2_S_3_ and WS_2_ implies that the electron transfer itself is sluggish, potentially due to surface adsorbate effects or limited active site regeneration, making it the RDS.^[^ ^45^ ^]^

**Figure 9:**
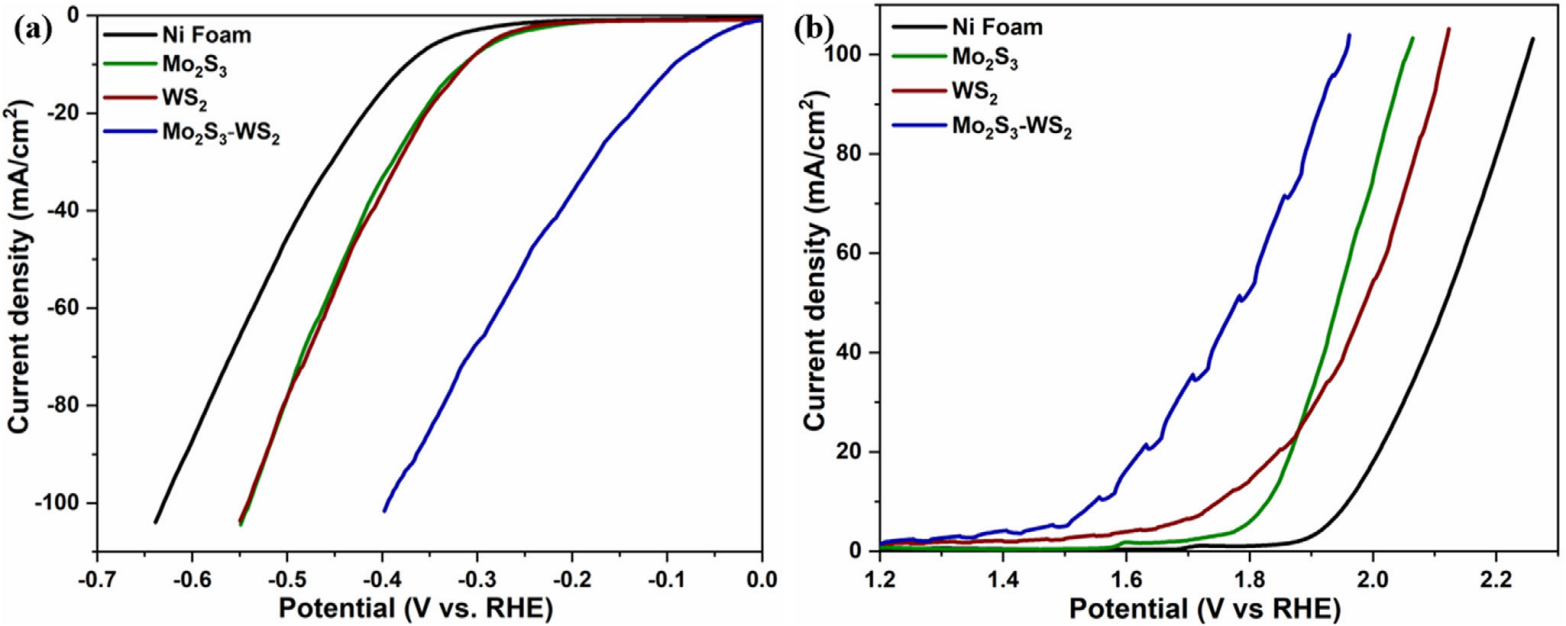
Photoelectrochemical a) HER and b) OER of Ni foam, Mo_2_S_3_, WS_2_ and Mo_2_S_3_‐WS_2_ catalysts on exposure toward 420 nm LED light source in 1 M KOH solution.

“Green hydrogen” production is noteworthy not only for its contribution to clean energy but also for its role in eliminating ozone‐depleting agents. In this context, investigating the promising photocatalytic activity of Mo_2_S_3_‐WS_2_ becomes crucial. To explore the photoelectrochemical activity of the catalyst, the LSV and photocurrent density (I versus t) measurements were employed under light illumination. As illustrated in Figure S10 (Supporting Information), the standard three‐electrode configuration serves as the experimental setup for the photoelectrochemical measurements. The Mo_2_S_3_‐WS_2_ catalyst exhibited remarkable photoelectrochemical performance for both HER and OER under illumination, highlighting its substantial potential for renewable hydrogen generation. When exposed to a pulsed light source (420 nm LED) with cycles of 10 s on and 5 s off, the catalyst achieved exceptionally low η, 92 mV for HER and 310 mV for OER at a current density of 10 mA cm^2^. These values are significantly lower than those measured for the individual catalysts (**Figure** **9**a,b).

**Figure 10:**
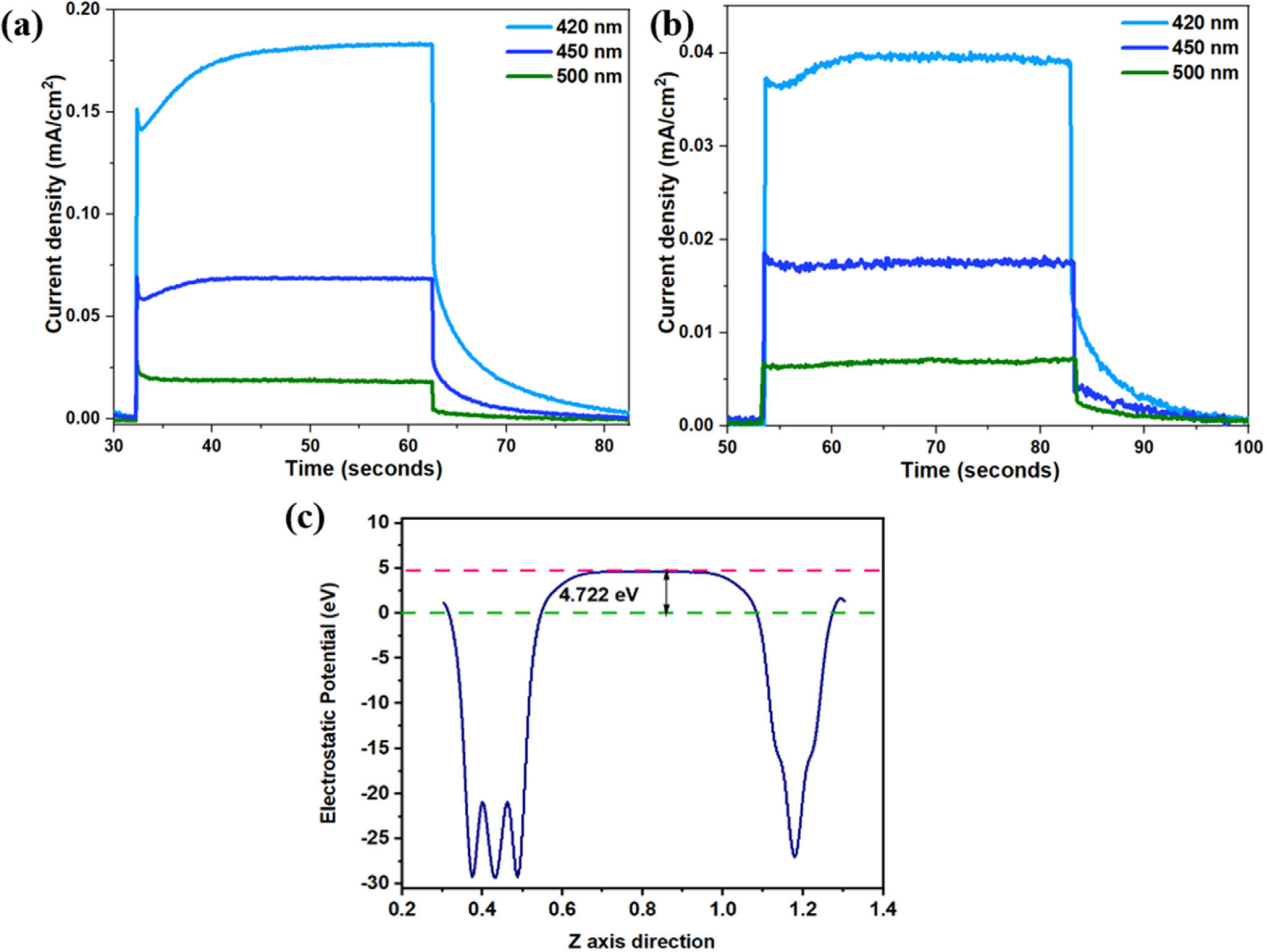
Comparison of photocurrent density response of Mo_2_S_3_‐WS_2_ catalyst measured on exposure toward different LED irradiation ranging from 420 to 500 nm with 20 s on/off pulses at bias voltage of a) +1.6 V versus RHE, b) −0.15 V versus RHE and c) work function of Mo_2_S_3_‐ WS_2_.

The photoelectrochemical performance of the Mo_2_S_3_‐WS_2_ sample was evaluated by measuring the photocurrent density across a range of light wavelengths (420, 450, and 500 nm) under both anodic (**Figure** **10**a) and cathodic illumination conditions (Figure 10b). The photocurrent demonstrated a stable preference for the anodic response, achieving a maximum density of 0.18 mA cm^2^, in contrast to a maximum of 0.04 mA cm^2^ observed under cathodic conditions. Illumination at 420 nm yielded optimal photogenerated electron transfer efficiency, suggesting that the band gap of the material aligns well with the photon energy at this wavelength. This alignment facilitates efficient light absorption, promotes charge carrier generation, and supports the effective generation and separation of photoexcited electrons within the material. The photocurrent response exhibits a gradual decline when illuminated with 450 and 500 nm LED sources. The stability of Mo_2_S_3_‐WS_2_ is a crucial feature for potential applications; notably, it maintains exceptional stability under electrochemical conditions and remains stable even after prolonged illumination. This durability is further demonstrated in Figure S11a,b (Supporting Information), where the photocurrent remains stable for over 2200 s. The minor reduction in current density during these measurements is likely due to partial material detachment in the solution. Additionally, the fast response and recovery times, shown in Figure S12 (Supporting Information), suggest efficient separation and migration of photogenerated charges, likely enhanced by an interfacial electric field within the composite.

**Figure 11:**
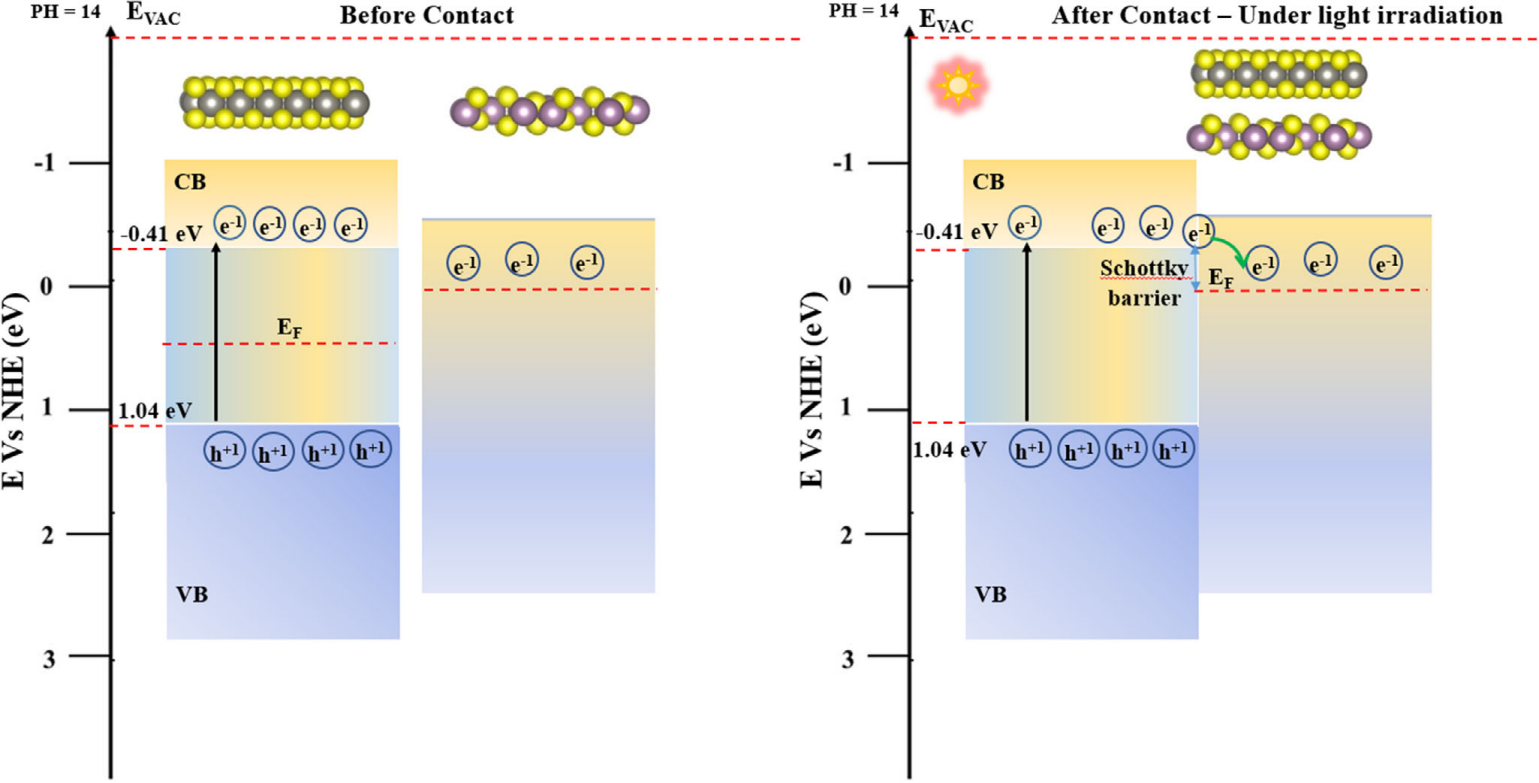
Photocatalytic mechanism of the Mo_2_S_3_‐WS_2_ heterostructure under simulated light irradiation.

In addition, in this study, density functional theory (DFT) calculations were used to investigate the electrostatic potentials and work functions of the Mo_2_S_3_‐WS_2_ heterostructure (Figure 10c), aiming to elucidate the charge transfer mechanism and the thermodynamic stability of electrons at the interface. The work function (Φ), being defined as the minimum energy required to remove an electron from the surface of the material, indicates its electron binding affinity and is determined by the difference between the vacuum level (E_vac_) and the Fermi level (E_F_). The work function of Mo_2_S_3_‐WS_2_ was calculated to be 4.722 eV and this value plays a critical role in dictating the band alignment and charge transfer at the heterojunction between WS_2_ and Mo_2_S_3_. By integrating information from UV–vis spectroscopy and DFT calculations, the proposed photocatalytic mechanism for Mo_2_S_3_‐WS_2_ composites can be comprehensively elucidated, shedding light on the key factors influencing their photocatalytic performance. Based on the analysis mentioned above, **Figure** **11**, likely illustrates the band‐alignment of the Mo_2_S_3_‐WS_2_ interface and the Schottky barrier, helping to visualize the proposed mechanism. The remarkable photocatalytic performance of the Mo_2_S_3_/WS_2_ composite relies on the formation of a favorable Schottky junction at the heterointerface. This arises from the intrinsic characteristics of the constituent materials. Mo_2_S_3_ possesses metallic characteristics, leading to a lower Fermi level compared to WS_2_. Under light illumination, this inherent electron‐capturing ability of Mo_2_S_3_ serves as a driving force for the transfer of photogenerated electrons from the excited WS_2_ to Mo_2_S_3_, creating photoinduced holes in the VB of WS_2_. This charge transfer process continues until the Fermi levels of both materials reach equilibrium, resulting in the formation of a Schottky barrier near the heterointerface.

This photoexcitation event is a crucial step in photocatalysis but suffers from inherent limitations due to electron‐hole recombination.^[^ ^46^ ^]^ However, the introduction of Mo_2_S_3_ facilitates the critical spatial separation of these photogenerated charges. The formation of a Schottky barrier at the interface between Mo_2_S_3_ and WS_2_ plays a critical role in promoting efficient separation of photogenerated charge carriers. This arises from the inherent difference in their Fermi levels. The lower Fermi level of Mo_2_S_3_ facilitates the spontaneous transfer of photoexcited electrons from WS_2_ to Mo_2_S_3_, leading to the establishment of equilibrium Fermi levels across the interface. The interfacial Schottky barrier impedes the back‐transport of electrons from Mo_2_S_3_ to WS₂, promoting efficient separation of the photogenerated charge carriers. This efficient separation significantly diminishes the rate of electron‐hole recombination, maximizing the utilization of photogenerated charges. Consequently, the isolated electrons in Mo_2_S_3_ can readily engage in reduction reactions, such as hydrogen evolution from protons (H⁺). Simultaneously, holes (h⁺) residing on the WS_2_ valence band effectively oxidize water molecules (H_2_O) into oxygen. This effective separation significantly improves the efficiency of both hydrogen evolution and oxygen evolution reactions. Overall, the incorporation of Mo_2_S_3_ into the photocatalytic system demonstrably enhances the separation of photogenerated carriers, leading to a substantial improvement in the overall photocatalytic performance.

Furthermore, the 2D morphology of Mo_2_S_3_ and WS_2_ further facilitates efficient electron transport within the composite. The resulting spatial separation of photogenerated electron‐hole pairs is effectively confirmed by electrochemical impedance spectroscopy (EIS) measurements. The trapped electrons on Mo_2_S_3_ then participate in the reduction reaction, reducing protons (H⁺) to molecular hydrogen (H₂). Simultaneously, holes (h⁺) residing on the WS_2_ valence band oxidize water molecules (H_2_O), generating hydroxyl radicals (‐OH) as intermediates, further promoting O_2_ evolution. In essence, the formation of a Schottky junction, attributed to the electron‐capturing ability of metallic Mo_2_S_3_ is the cornerstone to the enhanced photocatalytic activity observed in the composite.

**Figure 12:**
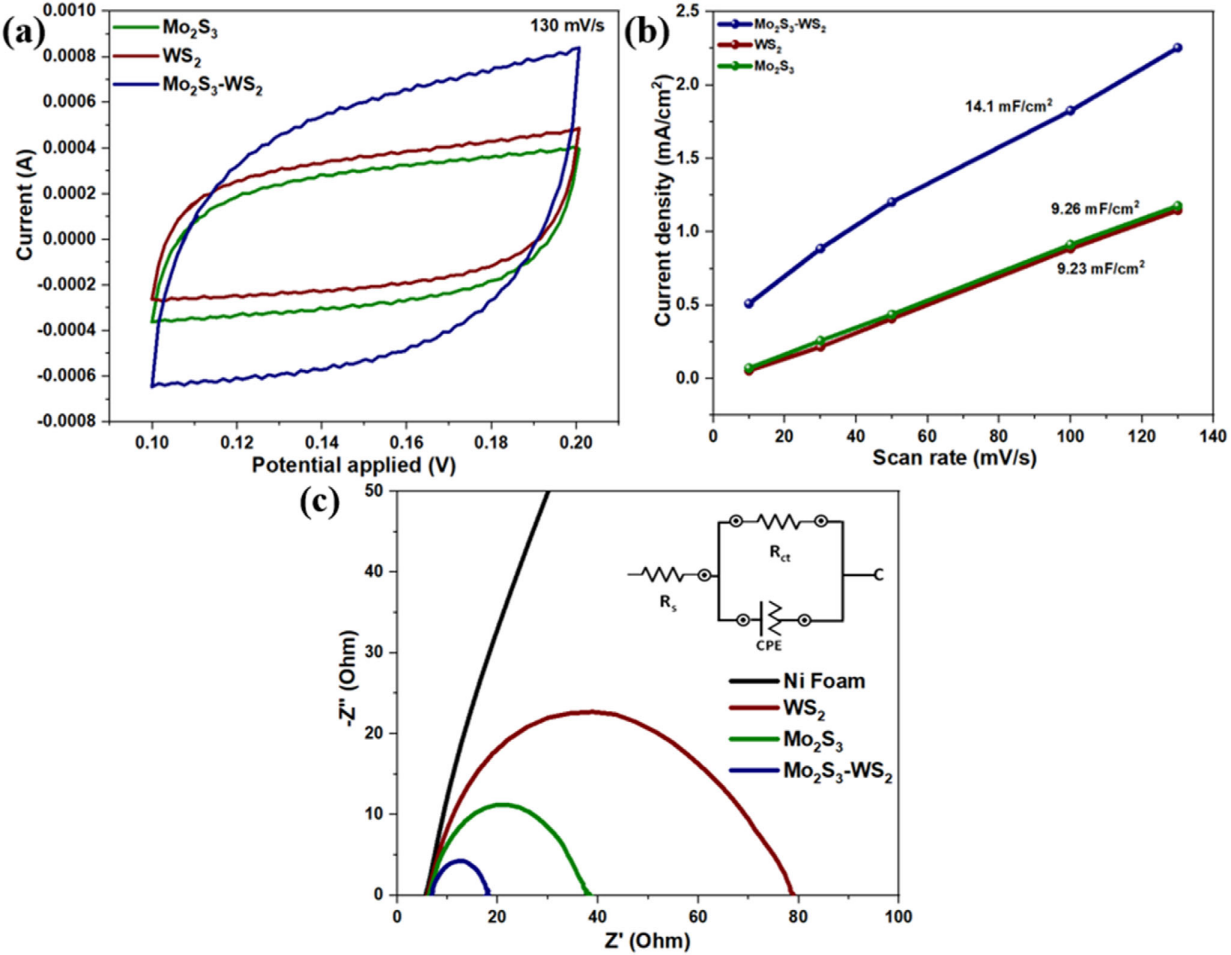
a) Cyclic voltammograms of Mo_2_S_3_, WS_2_, Mo_2_S_3_‐WS_2_ at a scan rate of 130 mV s^−1^, b) Scan rate dependence of the current densities at 1.07 V versus RHE of Mo_2_S_3_, WS_2_ and Mo_2_S_3_‐WS_2_ and c) electrochemical impedance spectra (EIS) of Ni foam, Mo_2_S_3_, WS_2_, Mo_2_S_3_‐WS_2_.

The electrochemical capacitances and the EIS analysis method serve as two crucial parameters for comprehensively assessing the catalytic activity and reaction kinetics at the electrode/electrolyte interface. The electrochemical double‐layer capacitances (C_dl_) were measured using the cyclic voltammetry (CV) technique at different scan rates (**Figure** **12**a; Figure S13, Supporting Information). The C_dl_ value of Mo_2_S_3_‐WS_2_ is measured at 14.1 mF cm^2^, surpassing those of Mo_2_S_3_ (9.26 mF cm^2^) and WS_2_ (9.23 mF cm^2^), as shown in Figure 12b. This suggests a more extensive electrocatalytic surface area with abundant active sites. Furthermore, the EIS analysis demonstrated the lowest transfer resistance (*R*_ct_) of 18.1 Ω for Mo_2_S_3_‐WS_2_, indicating efficient separation of photogenerated electron‐hole (e⁻/h⁺) pairs signifying relatively rapid charge transfer and high reaction kinetics (Figure 12c). This observation aligns well with the small Tafel slope and low η of Mo_2_S_3_‐WS_2_, highlighting the multi‐heterogeneous interfaces of Mo_2_S_3_‐WS_2_, which offer an extensive electrocatalytic surface area and expose abundant catalytic active sites, thereby enhancing the overall catalytic activity.

The formation of well‐defined heterointerfaces is critical for enhancing charge separation and interfacial charge transfer in heterostructured systems. Studies have demonstrated that engineered heterojunctions can significantly influence the electronic structure, catalytic activity and overall stability of the composite materials, leading to remarkable improvements in the electrochemical and photocatalytic performance.^[^ ^47^, ^48^, ^49^, ^50^, ^51^ ^]^ The electrochemical characterization of Mo_2_S_3_‐WS_2_ nanocomposite unequivocally demonstrates that its superior electro and photoelectrochemical performance stems from its unique 2D nanostructured morphology with different lateral dimensions. These uniformly distributed nanosheets exhibit a hierarchical architecture, resulting in a high specific surface area accentuated with abundant edge‐exposed active sites. This optimized morphology further supported the formation of a beneficial heterojunction, which is found to efficiently separate and transfer photogenerated charges, thus minimizing detrimental recombination losses.^[^ ^52^, ^53^ ^]^ Additionally, the plethora of edge sites on the 2D nanosheets acts as preferential centers for the target electro‐ and photochemical reactions. This, in synergistic interplay with the presence of the aforementioned heterojunction, leads to a significant optimization of the overall electro‐photocatalytic activity of Mo_2_S_3_‐WS_2_.

**Figure 13:**
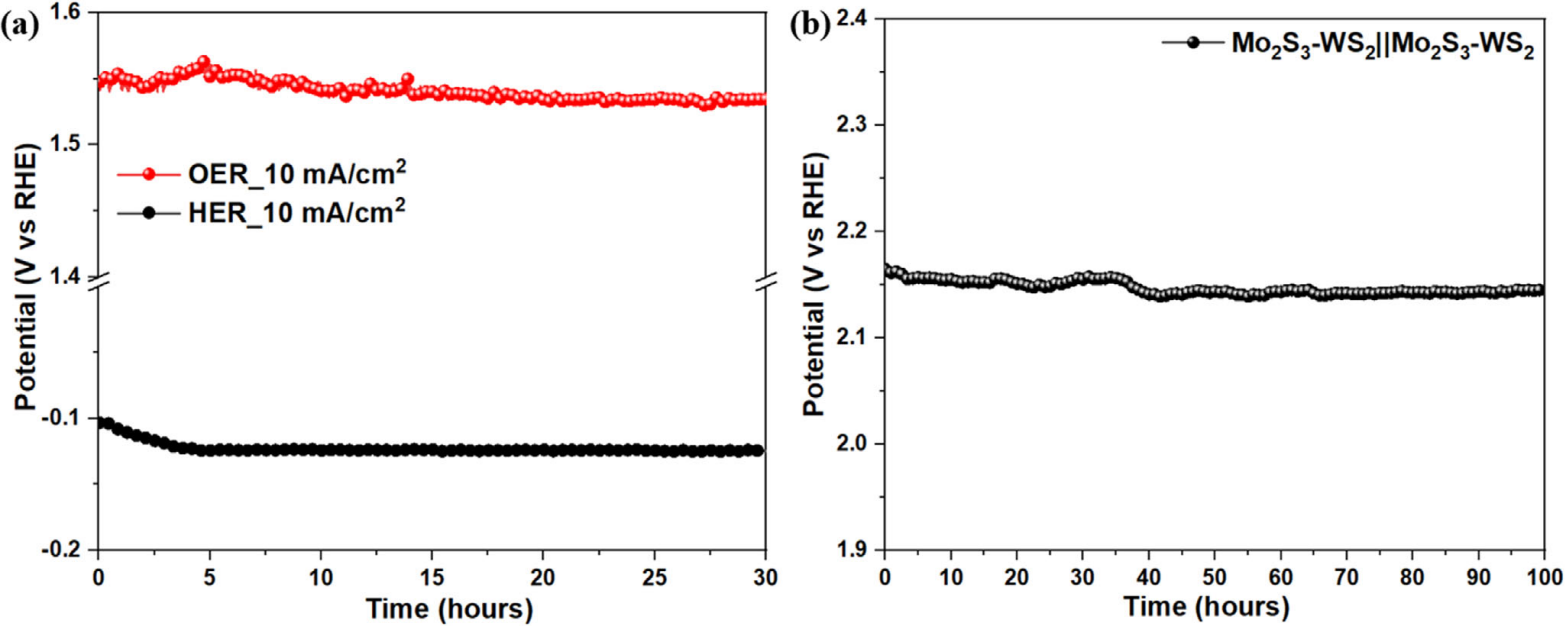
Chronopotentiometry plot of a) Mo_2_S_3_‐WS_2_ for cathodic and anodic potential at a constant current density of 10 mA cm^2^ for 30 h and b) Mo_2_S_3_‐WS_2_||Mo_2_S_3_‐WS_2_ maintained at a constant current density of 100 mA cm^2^ for 100 h.

For advanced viability, the long‐term stability and durability of Mo_2_S_3_‐WS_2_ catalysts are of paramount importance. Therefore, the long‐term stability test was performed by chronopotentiometry and the catalyst maintained consistent anodic and cathodic potentials for 30 h at a current density of 10 mA cm^2^ (**Figure** **13**a) with an average Δη(OER‐HER) value of 0.374 mV. Leveraging the excellent bifunctional activity of Mo_2_S_3_‐WS_2_ nanostructures for HER and OER, we performed the stability test of Mo_2_S_3_‐WS_2_|| Mo_2_S_3_‐WS_2_ at a high current density of 100 mA cm^2^ over 100 h (Figure 13b). The electrode demonstrated commendable stability during the 100 h test, experiencing negligible reduction in potential of 0.01% (≈20 mV). Furthermore, compared to several Mo_2_S_3_ and WS_2_ based nanocatalysts as presented in Table S1 (Supporting Information), Mo_2_S_3_‐WS_2_ catalyst showcases itself as an excellent bifunctional catalyst with improved catalytic activities.

To assess the stability, we conducted post‐characterizations of the catalyst after subjecting it to stability tests at various time intervals. Figure S14a (Supporting Information) presents STEM images and Figure S14b (Supporting Information) the SEM images of Mo_2_S_3_‐WS_2_ catalyst after a 30 h stability test. Additionally, the STEM‐EDX elemental mapping performed after the stability test also confirmed the presence of Mo, W, and S species (Figure S15, Supporting Information). Figure S16 (Supporting Information) indicates that even after subjecting the Mo_2_S_3_‐WS_2_ catalyst to a 30 h stability test, the Mo 3d, W 4f and S 2p XPS spectra exhibit negligible shifts. The stability of the nanomaterials was also validated through Raman measurements, where the materials maintained their spectra with negligible change (Figure S17, Supporting Information). Notably, even after performing the stability measurement for 30 h, Mo_2_S_3_‐WS_2_ catalyst maintained a small *R*_ct_ value of 22.3 Ω (Figure S18, Supporting Information). This suggests that the catalyst maintains its structural integrity and chemical composition under prolonged testing conditions. Through a strategic nanoengineering approach, we successfully tailored the as‐prepared Mo_2_S_3_‐WS_2_ catalyst to possess distinguishable photoelectrochemical performance toward efficient and stable H_2_ production.

**Figure 14:**
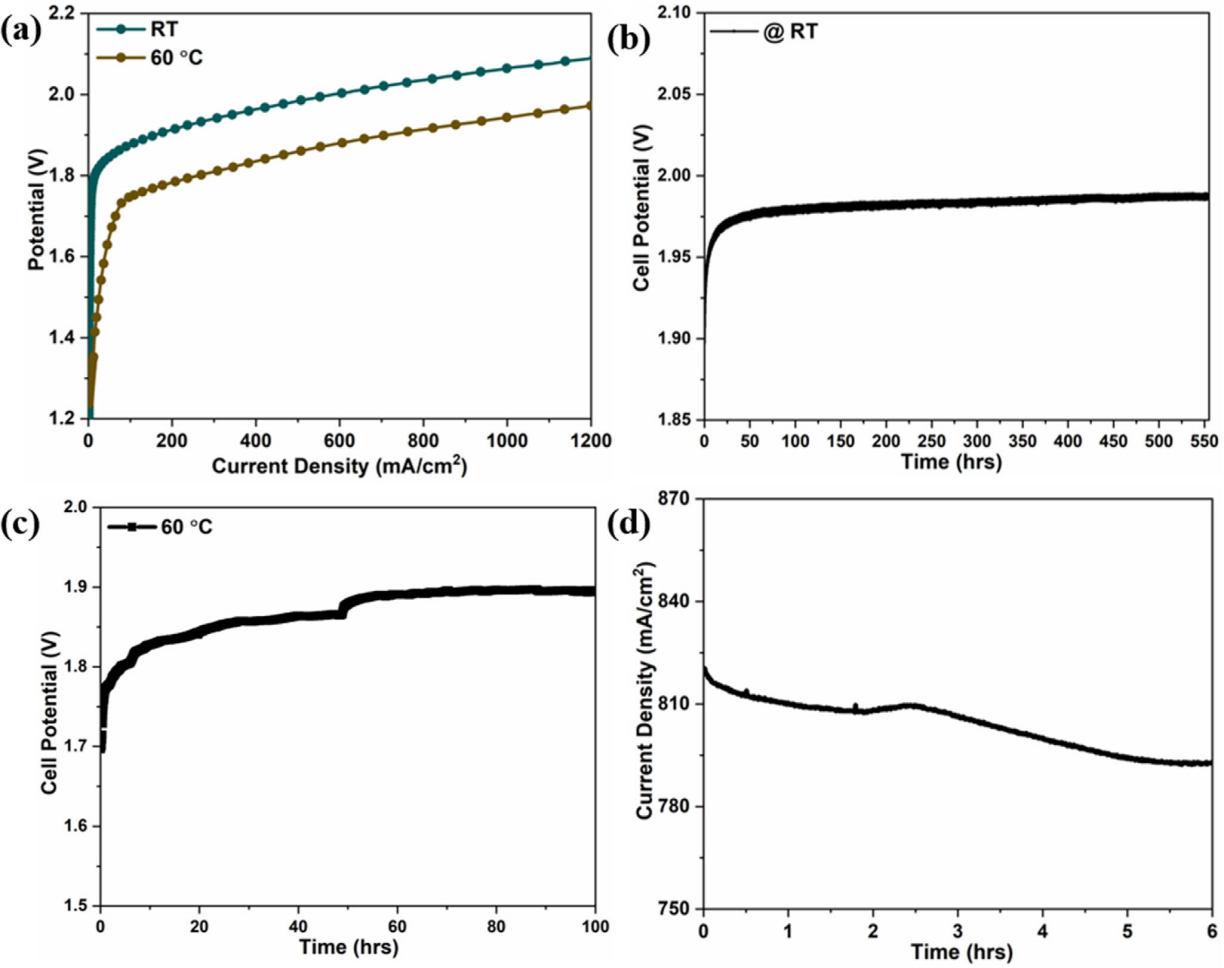
a) Polarization curves illustrating the performance of a zero‐gap alkaline water electrolyzer (Mo_2_S_3_‐WS_2_/NF || Mo_2_S_3_‐WS_2_/NF) in 6 M KOH at room temperature (RT) and 60 °C, Long‐term stability test demonstrating the durability of a zero‐gap alkaline water electrolyzer under continuous operation at 0.5 mA cm^2^ in 6 M KOH at b) RT and c) 60 °C, d) Chronoamperometric test of the electrolyzer operated at a constant cell voltage of 2 V in 6 M KOH upon integrating with solar panel.

The development of electrocatalysts with robust performance under industrial conditions, characterized by high current density, elevated temperature, and highly alkaline conditions, is crucial for industrial‐scale applications.^[^ ^54^ ^]^ Therefore, the Mo_2_S_3_‐WS_2_ catalyst was subjected to comprehensive testing using a zero‐gap single‐cell alkaline water electrolyzer using Mo_2_S_3_‐WS_2_ || Mo_2_S_3_‐WS_2_ as the cathode and anode with 6 M KOH electrolyte. As presented in **Figure** **14**a, in the zero‐gap assembly, the polarization curves of Mo_2_S_3_‐WS_2_ demonstrated high current densities of 0.5 and 1 A cm^2^ at low cell voltages of 1.98 and 2.07 V, respectively, at room temperature. The performance improved further when the operating temperature was increased to 60 °C, reaching 0.5 and 1 A cm^2^ at 1.86 and 1.95 V, corresponding to a voltage efficiency of 76% at 1 A cm^2^, which is attributed to enhanced electrode reactions. In addition, the assembly showed exceptional stability during a chronoamperometric test at 1.98 V for more than 500 h, showing outstanding operational stability (Figure 14b) with a H_2_ yield reaching 390 mL h^−1^. The stability of the Mo_2_S_3_‐WS_2_ catalyst was further confirmed by the chronoamperometric test at 60 °C as represented in (Figure 14c). Furthermore, by integrating the electrolyzer with a commercially available silicon solar cell, the feasibility of producing green hydrogen without carbon emissions using renewable electricity was demonstrated. The AWE equipped with Mo_2_S_3_‐WS_2_ || Mo_2_S_3_‐WS_2_ as cathode and anode was connected to the silicon solar cell and the performance of the integrated system was evaluated under outdoor environment (10:00 to 16:00 h). Under solar irradiation, the Mo_2_S_3_‐WS_2_ based electrolyzer achieved a current density of ≈0.8 A cm^2^ at an applied voltage of 2 V (Figure 14d). The Mo_2_S_3_‐WS_2_/NF catalyst demonstrated excellent long‐term stability, as evidenced by the minimal degradation observed in the polarization curves before and after the chronoamperometric test (Figure S19, Supporting Information). Furthermore, information from Figure S20 (Supporting Information) indicates that Mo_2_S_3_‐WS_2_ catalyst exhibits good stability under the tested conditions. Even after subjecting the Mo_2_S_3_‐WS_2_ catalyst to a stability test exceeding 500 h, the chemical state of the elements (Mo, W, and S) remains largely unchanged, suggesting that the active sites of the catalyst are not significantly altered. However, a decrease in sulfur concentration is observed as well as an oxidation of molybdenum in MoO_3_, but the fact that the overall oxidation state of W and S are preserved suggests that the observed decrease in sulfur concentration does not have a significant impact on catalytic performance. Overall, this work not only highlights the significant advantages of Mo_2_S_3_‐WS_2_ based catalysts, but also establishes a foundation for designing efficient 2D material‐based heterostructures for high‐performance electrolysis and the possibilities of integration with renewable power sources.

## 3 Conclusion

Our investigation successfully achieved the formation of the Mo_2_S_3_‐WS_2_ nanocomposite and demonstrated its superior photoelectrochemical activity for the generation of renewable hydrogen. This is convincingly corroborated by the remarkably low η achieved compared to those of pristine catalysts. The enhanced photocurrent density under illumination further confirms the efficient generation and separation of photoexcited charges within the material. Additionally, the Mo_2_S_3_‐WS_2_ composite likely benefits from an increased active surface area and reduced charge transfer resistance, which supports improved catalytic performance. The observed fast response‐recovery time during transient photocurrent measurements underscores efficient charge transfer processes within the catalyst. These comprehensive findings offer critical insights for optimizing photoactive materials in various renewable energy applications, including solar cells, photodetectors, and photocatalysis. Collectively, these findings establish Mo_2_S_3_‐WS_2_ as a promising candidate for advancing these technologies and contributing to the development of sustainable energy solutions. The study also introduces a novel approach to catalyst development by combining transition‐metal‐based 2D materials, paving the way for high‐performance and cost‐effective catalysts suitable for large‐scale alkaline water electrolysis.

Looking to the future, research could focus on the scalability and process optimization for the large‐scale synthesis of the Mo_2_S_3_‐WS_2_ composite to facilitate real‐world deployment. Moreover, incorporating this material into full‐cell configurations or photoelectrochemical systems powered by renewable energy sources would offer valuable insights into its potential for industrial hydrogen production. Further exploration of surface engineering or heteroatom doping strategies could also be pursued to fine‐tune its band structure, enhance catalytic efficiency, and extend its functionality across various pH conditions. These avenues present exciting opportunities for advancing 2D heterostructure‐based photocatalysts in next‐generation sustainable energy technologies.

## 4 Experimental Section

### Chemicals

Tungsten sulphide (WS_2_, 99.0%, Sigma–Aldrich, Czech Republic), molybdenum (99.999%, −100 mesh powder) was purchased from Shanghai Quken New Material Technology Co., China and sulfur (99.9999%, 2–6 mm granules) was purchased from Wuhan Xinrong New Material Co., China and utilized as received without additional purification.

### Synthesis of Mo_2_S_3_

The synthesis of Mo_2_S_3_ was performed by direct reaction of Mo and S elements in quartz ampoule, under high vacuum at high temperature (Figure S1, Supporting Information). Stoichiometric amounts of Mo and S corresponding to 10 g of Mo_2_S_3_ were placed in a quartz ampoule (30 × 150 mm) and sealed under high vacuum (<1 × 10^−3^ Pa, oil diffusion pump with LN_2_ trap). Subsequently, the ampoule was placed in muffle furnace and heated on 500 °C for 50 h, subsequently on 600 °C for 50 h and finally on 1000 °C for 72 h, with a heating and cooling rate of 1 °C min^−1^ resulting in a black powder.

a) *Exfoliation of WS_2_*: WS_2_ nanoflakes were exfoliated via high‐pressure microfluidic exfoliation using a pressure cell homogenizer (model FPG12800E‐F, 10 mL piston size, Homogenising Systems Ltd., UK).^[^ ^55^ ^]^ Initially, WS_2_ powder was dispersed in a water/isopropanol mixture (1:1 volumetric ratio) at a concentration of 5 mg mL^−1^. The dispersion was subjected to ultrasonication using a 100 W ultrasonic bath (Fisherbrand FB11203) for 30 min to ensure thorough mixing and dispersion of WS_2_ particles. The WS_2_ dispersion further underwent microfluidic exfoliation using a 0.1 mm diamond microfluidic channel. The dispersion was cycled through the microfluidic channel 30 times under a pressure of 200 MPa. This high‐pressure microfluidic exfoliation process facilitated the separation of WS_2_ layers, resulting in the production of exfoliated WS_2_ flakes.

b) *Preparation of Mo_2_S_3_‐WS_2_ composite*: Initially, 50 µL of the exfoliated WS_2_ suspension was taken in a glass vial and 30 mg of Mo_2_S_3_ powders were dispersed into this solution and subjected to ultra‐sonication for 5 h to improve the dispersion. The facile liquid exfoliation technique allowed the formation of Mo_2_S_3_‐WS_2_ composite with varied lateral dimension and served as the catalyst ink.

### Characterizations

The crystal structure and purity of the samples were investigated from X‐ray powder diffraction data obtained from Bruker D8 Discoverer diffractometer (Cu Kα radiation, λ = 0.15 418 nm, U = 40 kV and I = 40 mA), Bruker, Germany in Bragg–Brentano parafocusing geometry. The XRD patterns were recorded for scattering angle 2θ values over the range of 5 – 80° with a scanning step size of 0.05° and the data was examined using the High Score Plus 3.0 software. Raman spectra were acquired using an inVia Raman spectroscope (Renishaw, England). The instrument was configured in backscattering geometry and equipped with a charge‐coupled device (CCD) detector. A diode‐pumped solid state (DPSS) green laser with a wavelength of 532 nm, laser power of 0.75 milliwatts with 20× magnification objective was applied to the samples with an exposure time of 10 s, covering a spectral range from 100 – 700 cm^−1^. The sample morphology was investigated using a scanning electron microscope (SEM) equipped with a field‐emission gun (FEG) electron source (Tescan Maya, Czech Republic). The samples were mounted on carbon conductive tape prior to conducting SEM and energy dispersive X‐ray (EDX) spectroscopy measurements. These measurements were performed using a 20 kV electron beam. Energy dispersive X‐ray (EDX) spectroscopy, utilizing an SDD detector (X‐MaxN 80 TS) manufactured by Oxford Instruments, England was employed for elemental mapping. Further diluted suspension of samples was drop casted onto copper grid for high‐angle annular dark field scanning transmission electron microscopy (HAADF‐STEM‐EDX) analysis with 30 kV acceleration voltage. Transmission electron microscopy (TEM) and high‐resolution TEM (HRTEM) analysis were also performed for analyzing the morphology and structure of exfoliated material using a JEOL 2200 FS microscope (Jeol, Akishima, Japan) equipped with a TVIPS camera, operating at an accelerating voltage of 200 kV. The 3 µL from the prepared suspension were drop‐casted on a TEM grid (Cu, 300 mesh, Lacey Carbon from TED PELLA, Inc.) and dried in air. For analysis of surface composition and determination of oxidation states of various elements, X‐ray photoelectron spectroscopy (XPS) was employed. An ESCA Probe P spectrometer manufactured by Omicron Nanotechnology in Germany, equipped with a monochromatized Al Kα X‐ray source with an energy of 1486.7 electron volts (eV), was used for XPS analysis. The UV–vis spectroscopy measurements were conducted utilizing a LAMBDA 850+ UV–vis spectrophotometer manufactured by PerkinElmer, United States, covering a scan range from 250 to 800 nm.

### Theoretical Calculations

The density functional theory simulation of as identified 2D structure involving properties, structure optimization, band structure, density of states (DOS), optical properties (OP) were calculated using Material Studio‐CASTEP (Cambridge Sequential Total Energy Package code).^[^ ^56^, ^57^ ^]^ For the structural optimization, the ultrasoft pseudopotential was used to simulate the interaction between electron and ion cores in geometric structure optimization and single point energy calculation, and the PBE (Perdew Burke Ernzerhof) in generalized gradient approximation (GGA) was used to describe the exchange‐correlation function. Self‐consistent calculations are performed with the k‐point sampling of 4 × 2 × 2 with the convergence threshold for SCF tolerance was 2 × 10^−6^ eV atom^−1^ between two electronic steps, and the maximum force upon each atom was less than 0.01 eV Å^−1^. The cut off energy was set to 450 eV.

### Electro‐Photochemical Measurements

The electrochemical experiments, linear sweep voltammograms (LSV), chronoamperometry, chronopotentiometry, cyclic voltammograms (CV) and electrochemical impedance spectroscopy (EIS) were performed at room temperature using Metrohm Autolab PGSTAT204 electrochemical workstation (Utrecht, The Netherlands, NOVA Version 2.1.5). The electro‐ and photo‐ chemical measurements were performed using a three‐electrode configuration cell consisting of nickel foam (NF) coated with material as working electrode (WE), Hg/HgO reference electrode (RE) and a carbon rod as counter electrode. 1 M KOH solution serves as the electrolyte and the potentials were represented with respect to reversible hydrogen electrode (RHE) using the equation, E_RHE_ = E_Hg/HgO_ + 0.059×pH + E^0^_Hg/HgO_, where E_Hg/HgO_ is the measured potential versus the Hg/HgO, pH is the electrolyte pH and E^0^_Hg/HgO_ is the standard E_Hg/HgO_ electrode potential, corresponding to 0.098 V at 25 °C.

To assess the electrochemical activity of the nanostructures, the as‐prepared catalyst ink was coated onto a 1 cm^2^ nickel foam (NF) substrate with a mass loading of ≈5.5 mg cm^2^, followed by drying at 60 °C for 15 min. Polarization curves were recorded via linear sweep voltammetry (LSV) measurements, employing a scan rate of 5 millivolts per second (mV/s). The overpotential for HER was determined using the formula η = 0 – E_RHE_, where η represents overpotential in volts (V). In the context of OER, η was calculated using the equation η(V) = E_RHE_ – $E_{H_{2}O/O_{2}}$, where $E_{H_{2}O/O_{2}}$ is the thermodynamic potential value of the OER at 25 °C with a value of 1.23 V versus RHE. The Tafel slope (TS) was derived from the equation η = a + b log|j|, with η, a, b and j representing the overpotential, axis intercept, the TS, and the current density, respectively. Nyquist plots were generated from EIS measurements for at +1.6 V (versus RHE) across a frequency range of 0.1 Hz to 100 kHz with an amplitude of 10 mV. To evaluate the electrochemical capacitances of the catalyst, cyclic voltammetry (CV) was performed in the non‐Faradaic region at various scan rates (10, 30, 50, 100, and 130 mV s^−1^). The double‐layer capacitance (C_dl_) was determined from the slope of the linear fit between scan rates and the difference in anodic and cathodic current densities, based on the equation:

(1) $$C_{dl}=\frac{j_{a}-j_{c}}{2\upsilon }=\frac{j_{a}+\left|j_{c}\right|}{2\upsilon }=\frac{\Delta j}{2\upsilon }$$

where j_a_ and j_c_ are the anodic and cathodic current densities, respectively, measured at the midpoint of the selected potential window, and υ is the scan rate.

Chronopotentiometry measurements were performed for 30 h to determine the long‐term stability of the catalyst at a current density of 10 mA cm^2^. The overall water splitting efficiency was assessed using a two‐electrode configuration wherein both the electrodes were coated with Mo_2_S_3_‐WS_2_ catalysts on NF. Further, Mo_2_S_3_‐WS_2_/NF electrodes were integrated into a zero‐gap configuration within a single‐cell alkaline water electrolyzer having an active area of 25 cm^2^, using a commercial sustainion X37‐50 membrane served as the separator. The electrolyzer stack comprises of two end plates, gaskets, a cathode, a membrane and an anode. A symmetric electrolyte feed (6 M KOH) was circulated through both the anodic and cathodic chambers using a peristaltic pump at ambient and elevated temperatures (room temperature and 60 °C). Polarization curves were generated by sweeping the current density from 0 to 1 A cm^−2^, while simultaneously recording the corresponding cell voltages. Durability testing was also performed by running the electrolyzer at a constant current density of 0.5 A cm^2^ over 500 h. Additionally, an integrated system combining the electrolyzer with a solar panel was tested to explore the feasibility of sustainable energy‐driven electrolysis.

This file includes figures of synthesis of Mo_2_S_3_, X‐ray diffraction (XRD) of Mo_2_S_3_ and WS_2_ nanostructures, X‐ray photoelectron spectroscopy (XPS) spectra of Mo_2_S_3_ and WS_2_, energy dispersive X‐ray (EDX) spectra of Mo_2_S_3_, WS_2_ and Mo_2_S_3_‐WS_2_, scanning transmission electron microscopy (STEM) of Mo_2_S_3_‐WS_2_, experimental set‐up for photoelectrochemical measurement, photoresponse of Mo_2_S_3_‐WS_2_, Photocurrent response of Mo_2_S_3_‐WS_2_ catalyst measured on exposure toward 420 nm LED, Cyclic voltammograms of Mo_2_S_3_, WS_2_ and Mo_2_S_3_‐WS_2_, table comparing the catalytic activity, STEM of Mo_2_S_3_‐WS_2_ before and after electrochemical studies, SEM‐EDX, STEM‐EDX, XPS, Raman and EIS spectra of Mo_2_S_3_‐WS_2_ after electrochemical stability test for 30 h, polarization curves of Mo_2_S_3_‐WS_2_ based electrolyzer before and after stability test at room temperature and 60 °C and XPS spectra of Mo_2_S_3_‐WS_2_ based electrolyzer after stability test at room temperature over 500 h.

## Conflict of Interest

The authors declare no conflict of interest.

## Supporting information



Supporting Information

## Data Availability

The data that support the findings of this study are openly available in Zenodo at https://doi.org/10.5281/zenodo.14391089, reference number 14391089.
