# Supplementary material for: Unravelling the Electro‐Photocatalytic Water Splitting Capabilities of 2D‐Bifunctional Mo2S3‐WS2 Catalyst: Implications for Renewable Energy Platforms
Source: Small Methods. 2025 Jul 26;9(9):e00476. doi: 10.1002/smtd.202500476 (PMC12464802; doi:10.1002/smtd.202500476)
Supplement: Supplementary file 1 — Supporting Information [file SMTD-9-e00476-s001.pdf]

small methods

Supporting Information

for *Small Methods*, DOI 10.1002/smtd.202500476

Unravelling the Electro-Photocatalytic Water Splitting Capabilities of 2D-Bifunctional Mo_2S_3-WS_2 Catalyst: Implications for Renewable Energy Platforms

Levna Chacko, Amutha Subramani, Jakub Regner, Pradip Kumar Roy, Rui Gusmão, Roussin Lontio Fomekong, Shuangying Wei, Vlastimil Mazánek and Zdeněk Sofer**

Supporting Information

Unravelling the Electro-Photocatalytic Water Splitting Capabilities of 2D-Bifunctional Mo_2S_3-WS_2 Catalyst: Implications for Renewable Energy Platforms

Levna Chacko[1], Amutha Subramani[1], Jakub Regner[1], Pradip Kumar Roy[1,2], Rui Gusmão[1], Roussin Lontio Fomekong[1,3], Shuangying Wei[1], Vlastimil Mazánek[1], Zdeněk Sofer*[1]*

[1]*Department of Inorganic Chemistry, University of Chemistry and Technology Prague, Technicka 5, 16628 Prague 6, Czech Republic*

[2] *Centre of Excellence ENSEMBLE3 Sp. z o. o. Wolczynska Str. 133, 01-919 Warsaw, Poland*

[3] *Higher Teacher Training College, University of Yaounde I, P.O. BOX 47, Yaounde, Cameroon*

Corresponding authors: Levna Chacko, Zdeněk Sofer

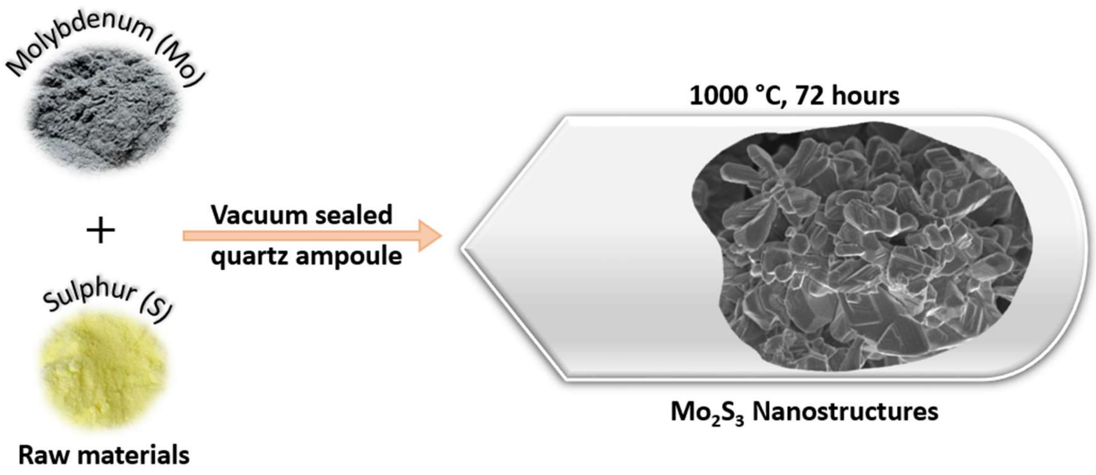

Figure S1: Schematic illustration of synthesis of Mo_2S_3 nanostructures.

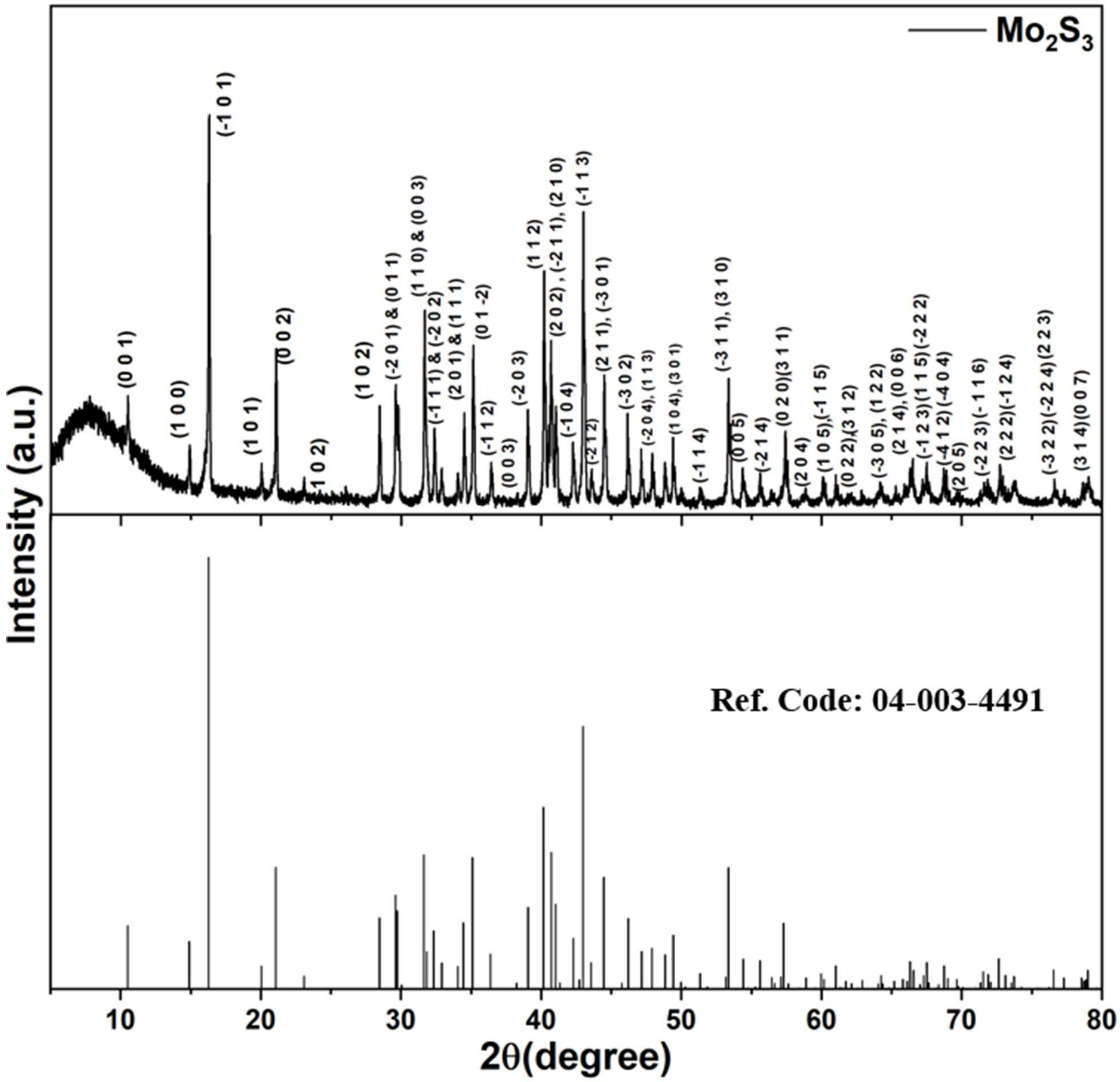

Figure S2: XRD of Mo_2S_3 nanostructures.

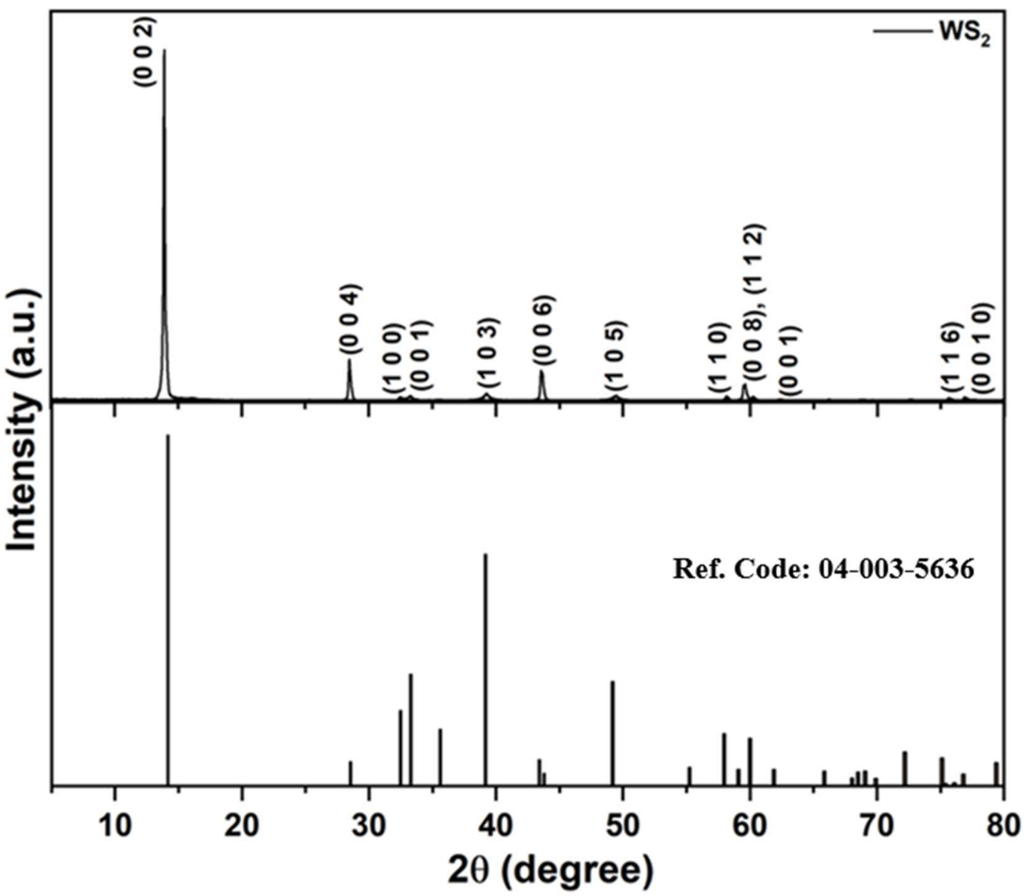

Figure S3: XRD of WS_2 nanostructures.

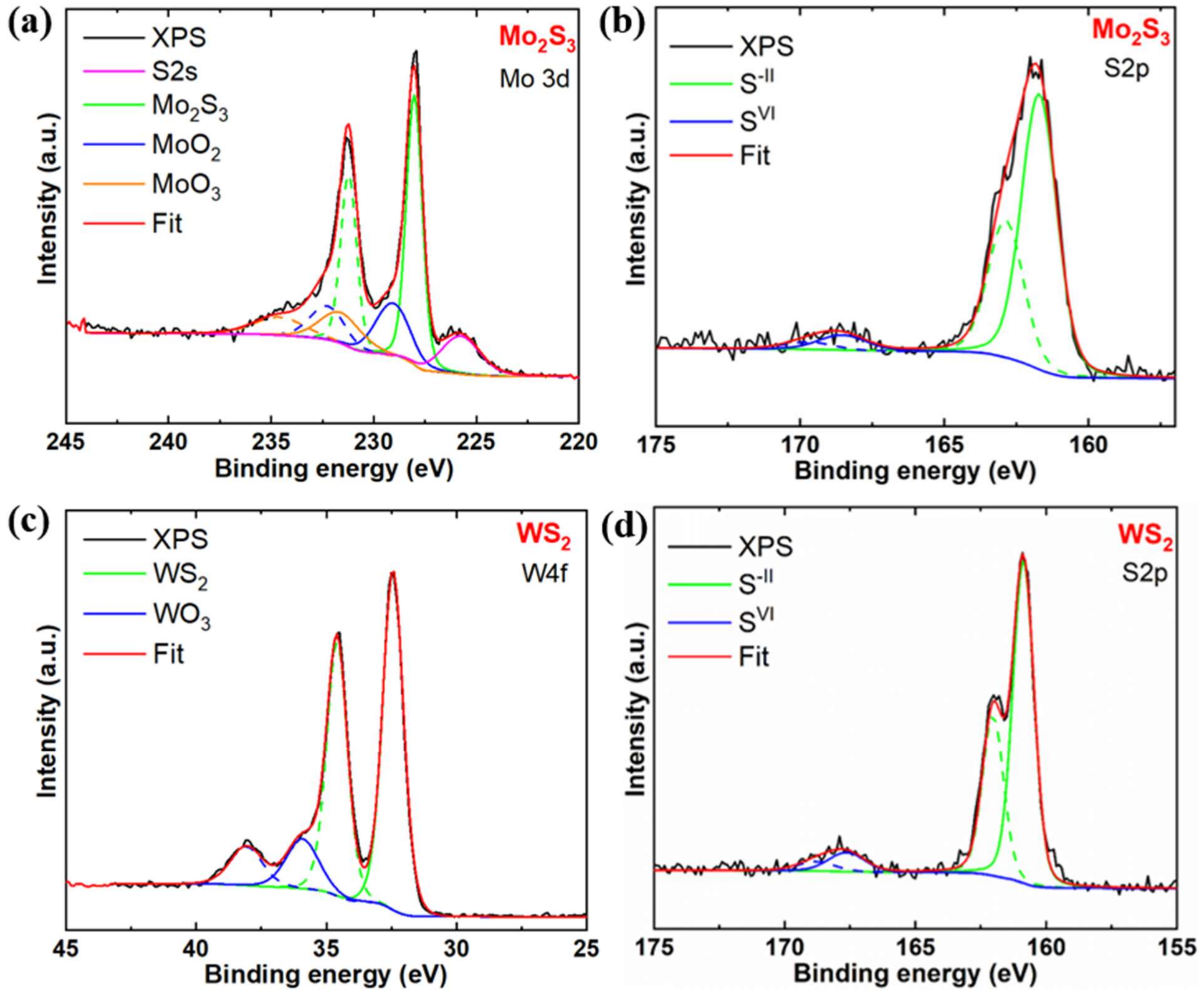

Figure S4: XPS high-resolution spectra of (a) Mo 3d, (b) S 2p of Mo_2S_3 and (c) W 4f, (d) S 2p of WS_2 nanostructures.

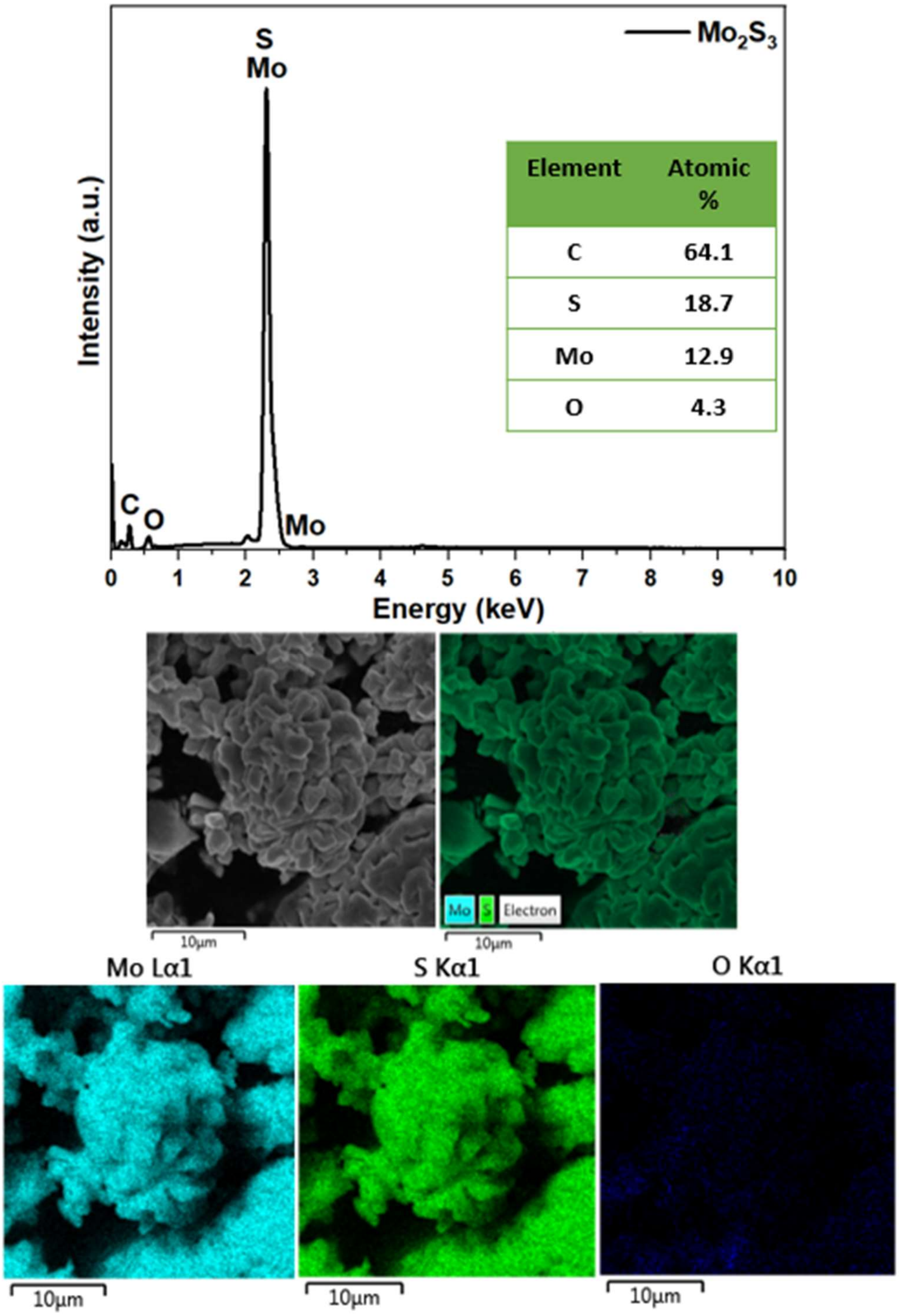

Figure S5: EDX spectrum and elemental mapping of Mo_2S_3.

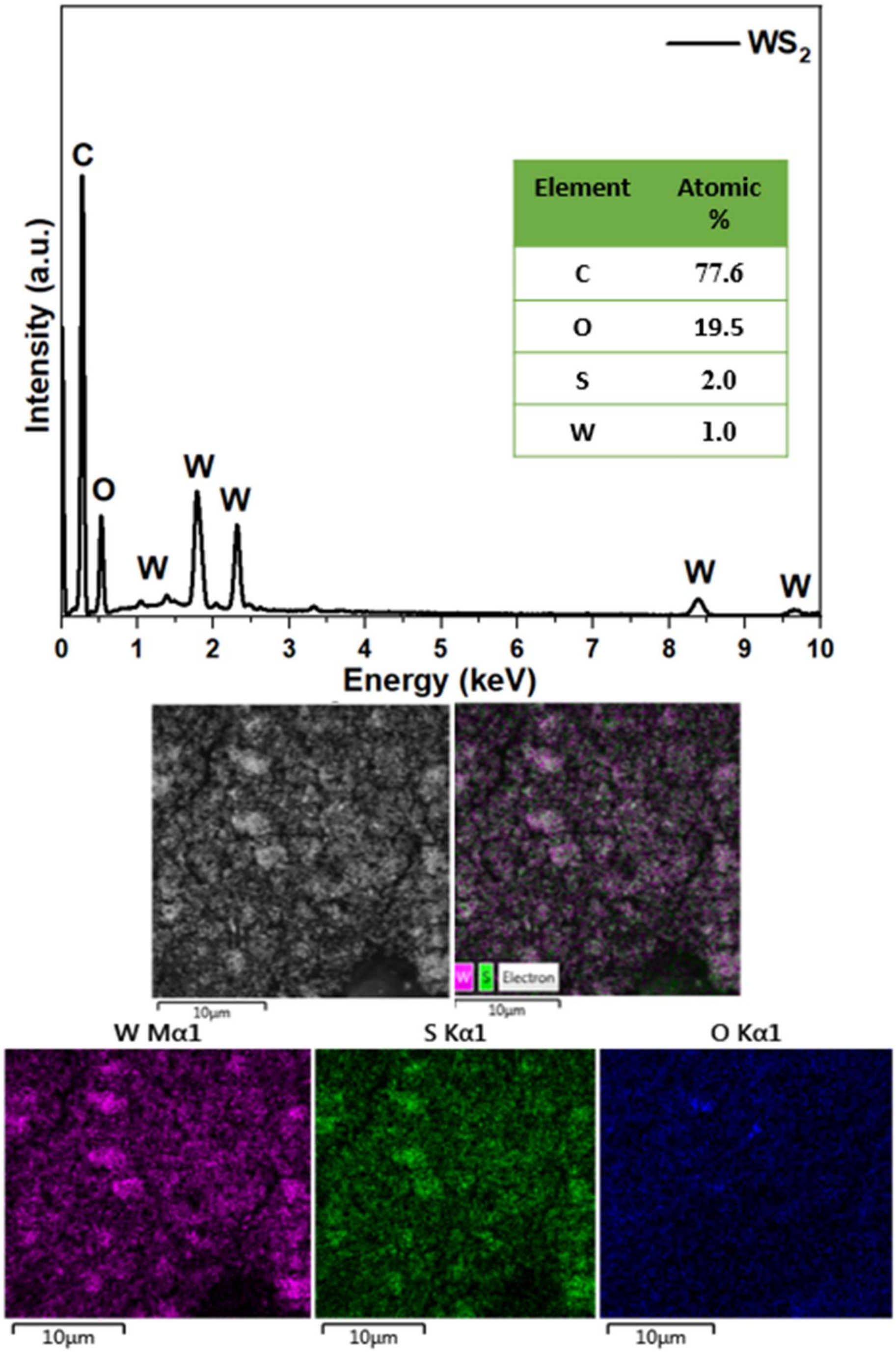

Figure S6: EDX spectrum and elemental mapping of WS_2.

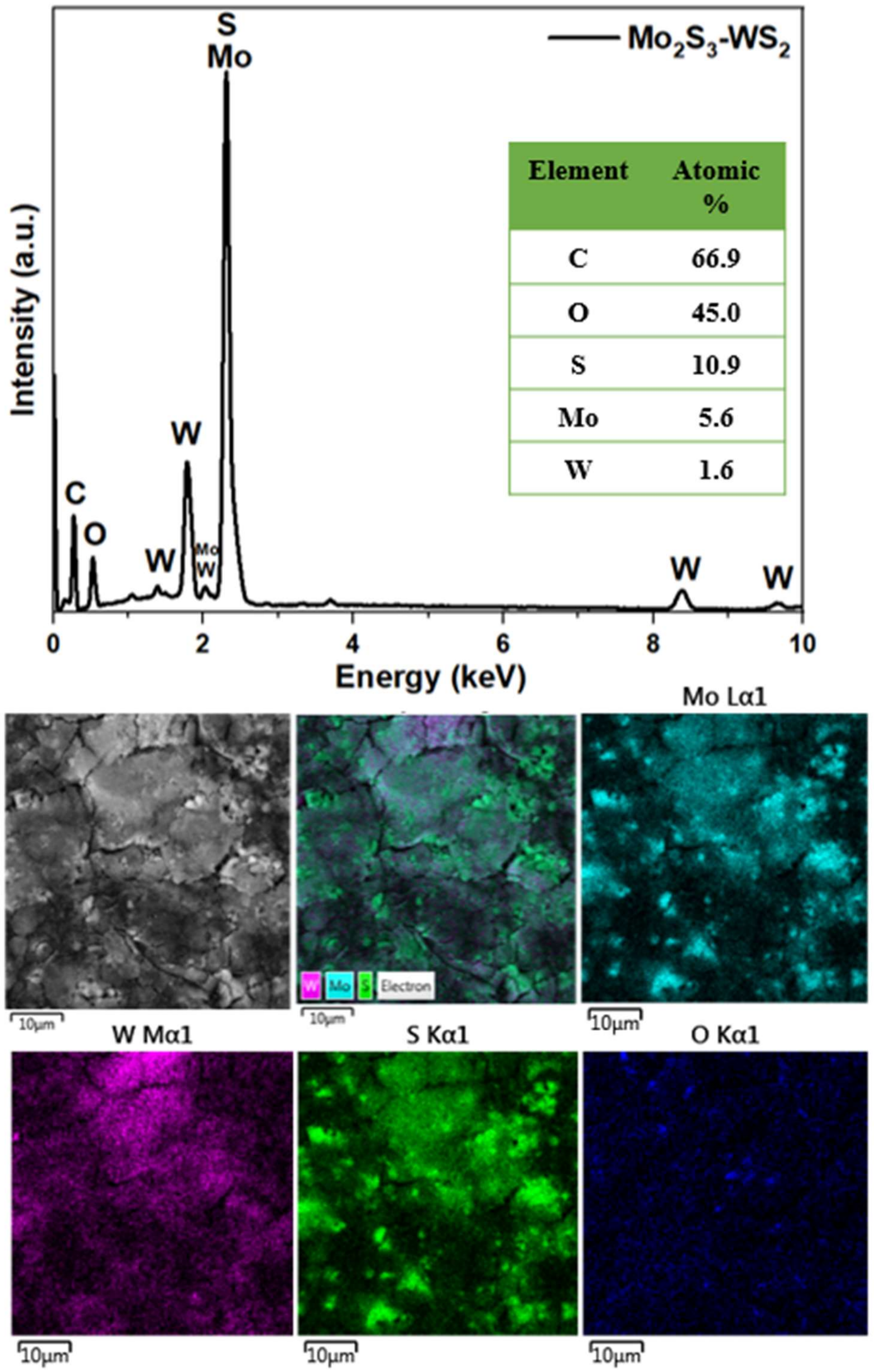

Figure S7: EDX spectrum and elemental mapping of Mo_2S_3-WS_2.

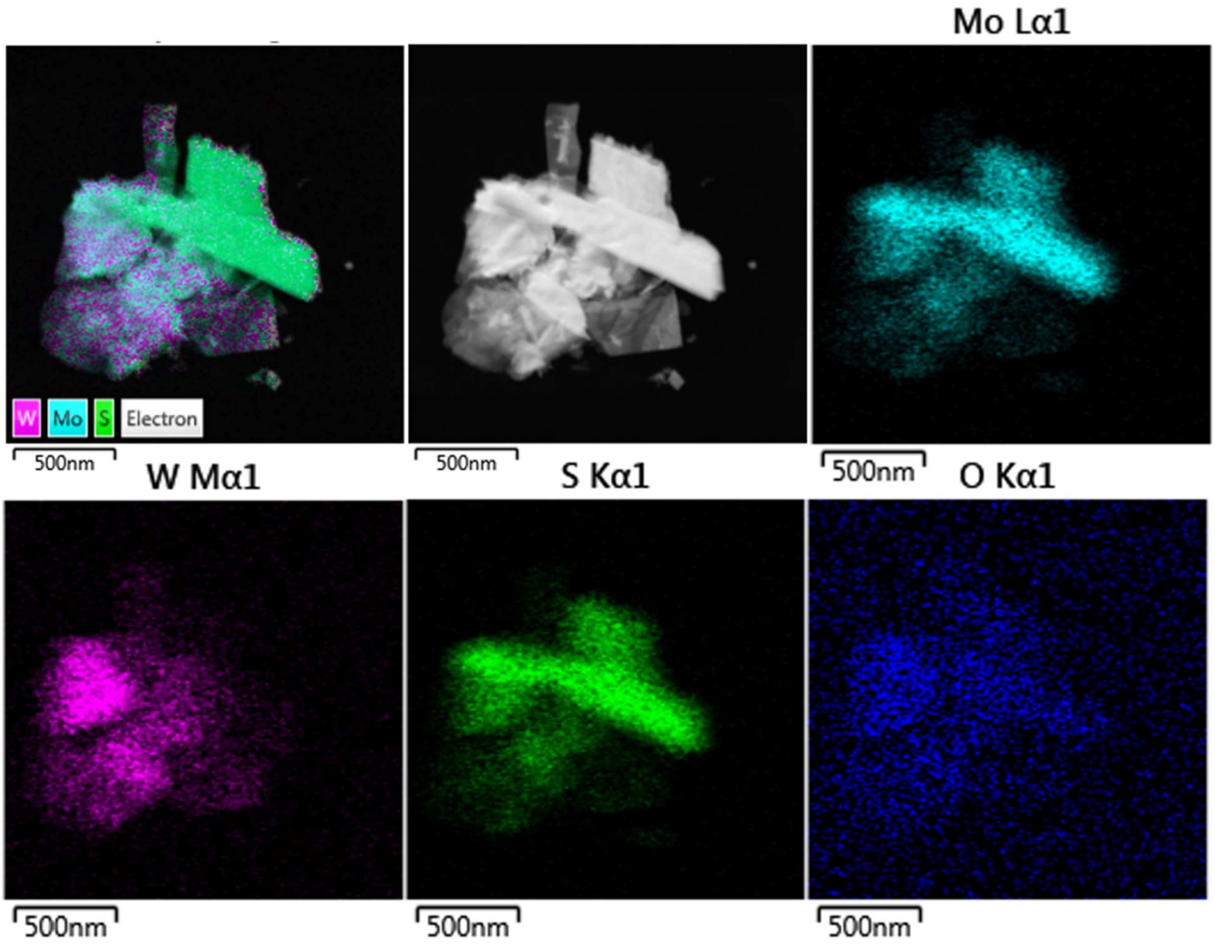

Figure S8: STEM-EDX mapping images of Mo_2S_3-WS_2.

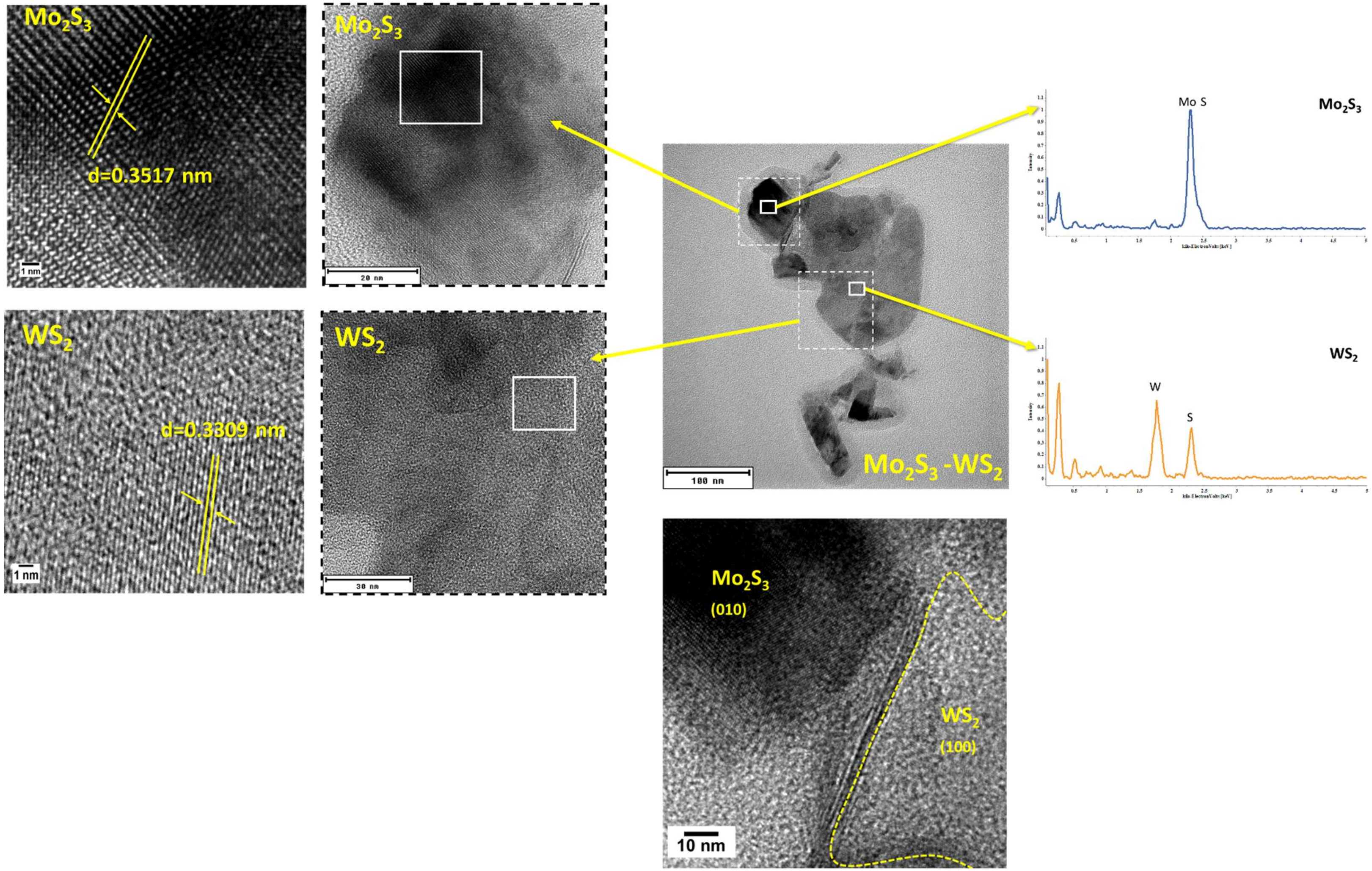

Figure S9. HR-TEM characterization of Mo_2S_3-WS_2 heterostructured composite. Center middle: HRTEM micrograph with analysis region delineated (squares). Right: EDX spectrum quantifying elemental distribution across of the zones indicating Mo_2S_3 or WS_2. Left: Selected crystallographic domain for interplanar d-spacing estimation by FFT analysis of lattice fringes. Center bellow: estimation of boundary demarcation (dotted line), with Mo_2S_3 (010) lattice on the and on the right 2H-WS_2 displaying (100) interplanar spacing.

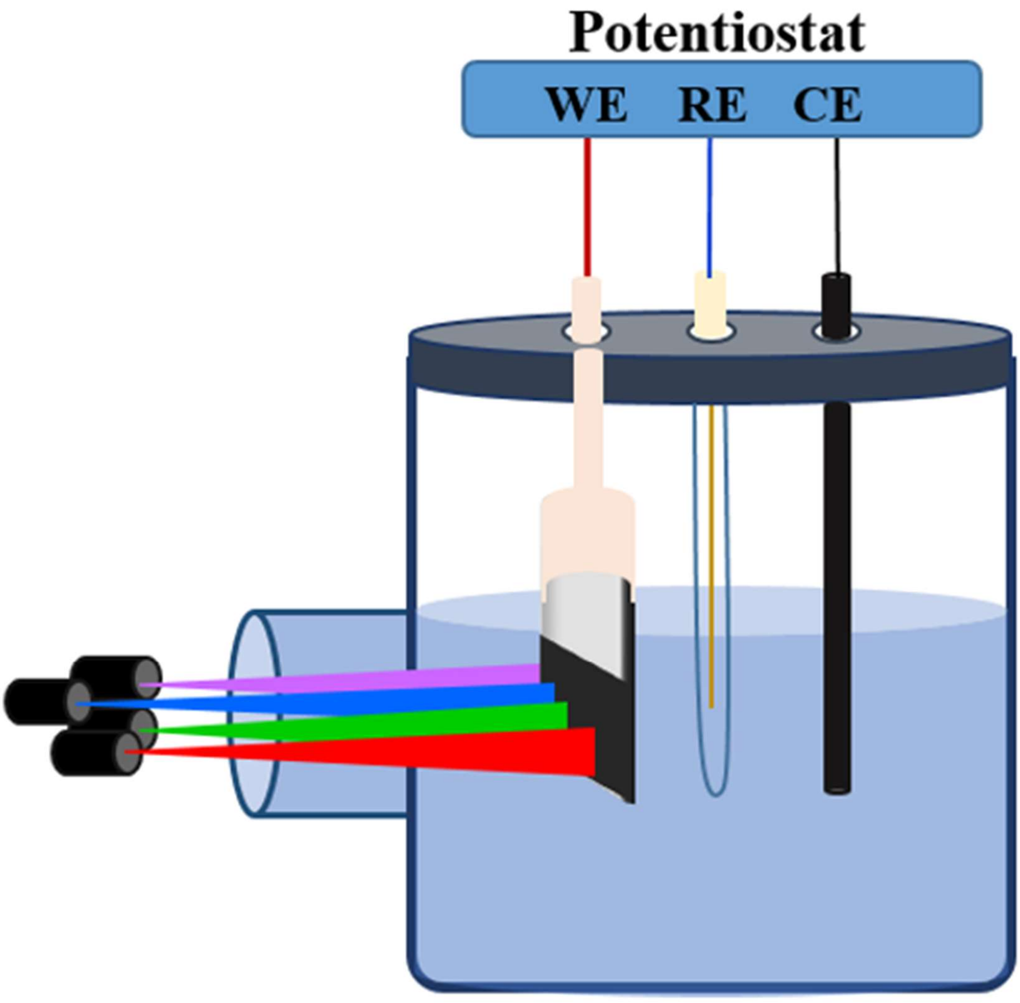

Figure S10: Experimental arrangement for photoelectrochemical measurement employing three electrode set-up.

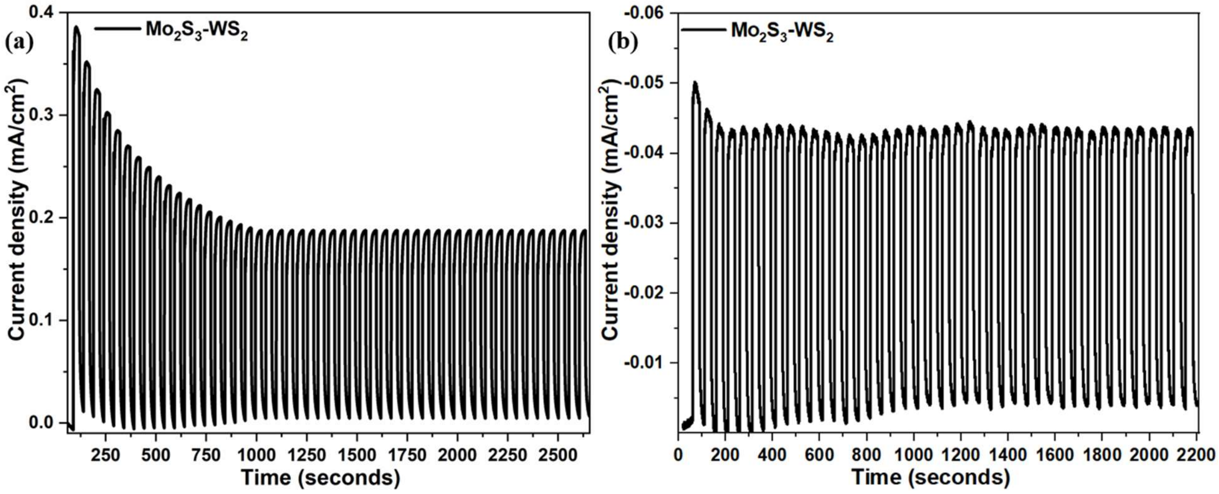

Figure S11: Stable photocurrent density response of Mo_2S_3-WS_2 catalyst measured on exposure towards 420 nm LED irradiation with 20s on/off pulses at bias voltage of (a) +1.6 V vs RHE, (b) -0.15 V vs RHE.

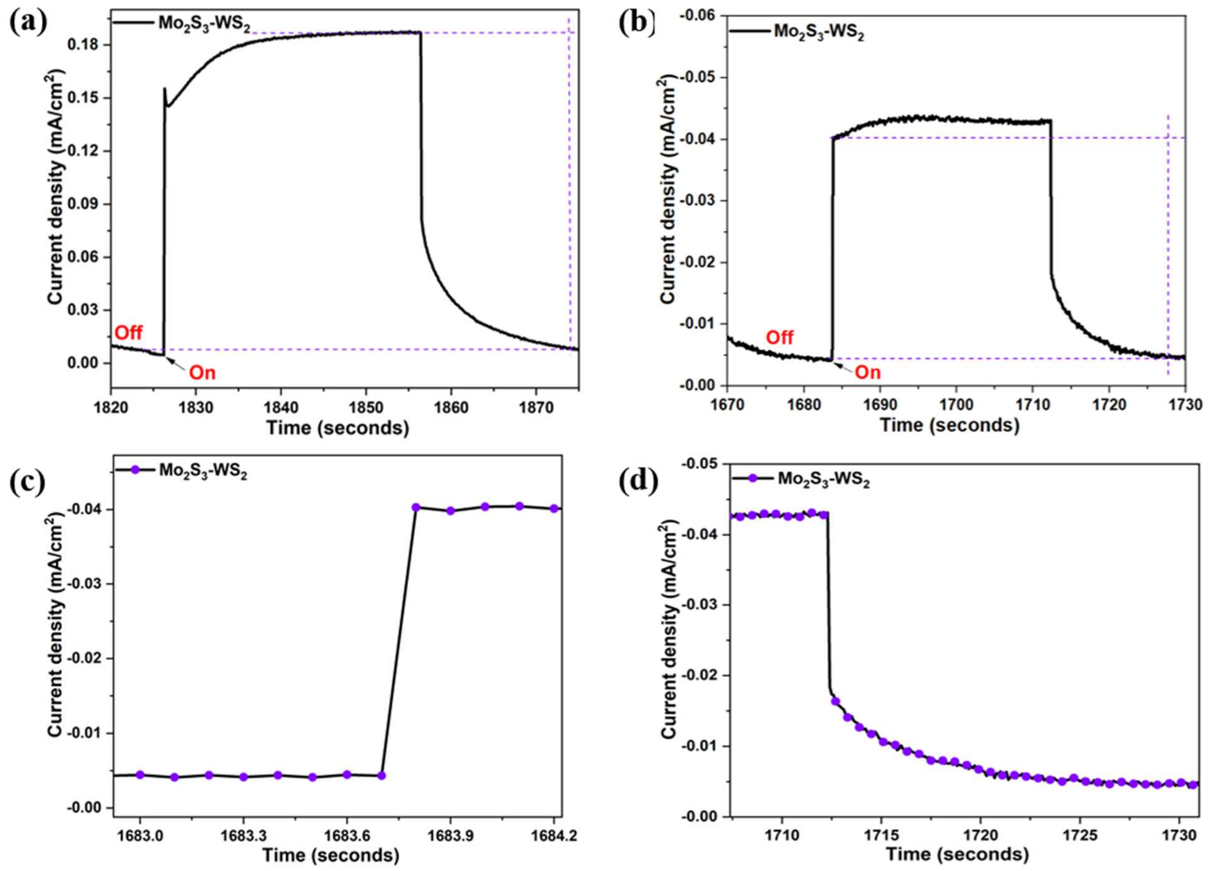

Figure S12: Photocurrent response of Mo_2S_3-WS_2 catalyst measured on exposure towards 420 nm LED on/off at bias voltage of (a) +1.6 V vs RHE, (b) -0.15 V vs RHE, and (c) response time and (d) recovery time of Mo_2S_3-WS_2 on exposure towards 420 nm LED at bias voltage -0.15 V vs RHE.

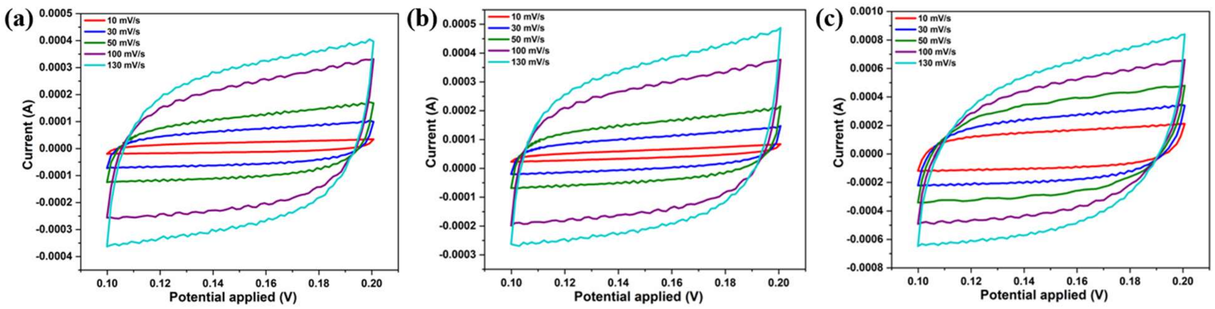

Figure S13: Cyclic voltammograms of (a) Mo_2S_3, (b) WS_2 and (c) Mo_2S_3-WS_2 at different scan rate ranging from 10 to 130 mV/s.

Table S1: Comparison of catalytic activity of various Mo_2S_3, MoS_2 and WS_2 based nanocatalysts.

**Photocatalyst**	**Electrolyte**	**HER Overpotential**	**OER Overpotential**	**Photocurrent**	**Stability**	**Ref**
M-NiS/Mo_2S_3 (M = Co, Fe, Ce and Bi)	1 M KOH	142 mV at 10 $mA.cm^{-2}$			1.574 V 50 mA. cm^{-2}	[1]
$Mo_2S_3/Bi_2O_2CO_3$	Na_2SO_4 solution	320 mV at 10 $mA.cm^{-2}$		< 1 $\mu A.cm^{-2}$		[2]
Mo_2S_3/NSCS-50	0.5 M H_2SO_4	106 mV at 10 $mA.cm^{-2}$				[3]
$CuInS_2/Mo_2S_3$				< 0.3 $\mu A.cm^{-2}$		[4]
Mo_2S_3/BiOBr	Gatifloxacin & tetracycline hydrochloride			0.7 $\mu A.cm^{-2}$		[5]
Cu_9S_5/Mo_2S_3/NF	1M KOH	96 mV at 10 $mA.cm^{-2}$	224 mV at $mA.cm^{-2}$		1.55 V at 10 $mA.cm^{-2}$	[6]
Mo_2S_3/MoP_2	0.25 M of H_2SO_4	250 mV at 10 $mA.cm^{-2}$				[7]
$WS_2/Zn_3In_2S_6$	0.35 M $Na_2S\cdot 9H_2O/NaH_2PO_2$			~ 0.1 $mA.cm^{-2}$		[8]
1T-WS_2/NCN	Triethanolamine			~ 2.5 $\mu A.cm^{-2}$		[9]
Ni-CdS/1T-WS_2	Lactic acid			< 20 $\mu A.cm^{-2}$		[10]
Cu-$ZnIn_2S_4/WO_3/WS_2$	0.1 M ascorbic acid			< 0.5 $\mu A.cm^{-2}$		[11]
$Ti_3C_2T_x$/1T-WS_2/CdS	Lactic acid	~ - 0.8 V at 10 $mA.cm^{-2}$		< 80 $\mu A.cm^{-2}$		[12]
MoS_2/WS_2/CdS	0.5 $mol\cdot L^{-1}$ Na_2SO_4	~ - 0.85 V at 10 $mA.cm^{-2}$		~ 14 $\mu A.cm^{-2}$		[13]
MnO_x-CdS-MoS_2	Lactic acid			~ 6 $\mu A.cm^{-2}$		[14]

Mo_2S_3-WS_2	1M KOH	92 mV at 10 mA.cm^{-2}	310 mV at 10 mA.cm^{-2}	~ 0.2 mA.cm^{-2}	~2.15 V at 100 mA.cm^{-2}	(This work)

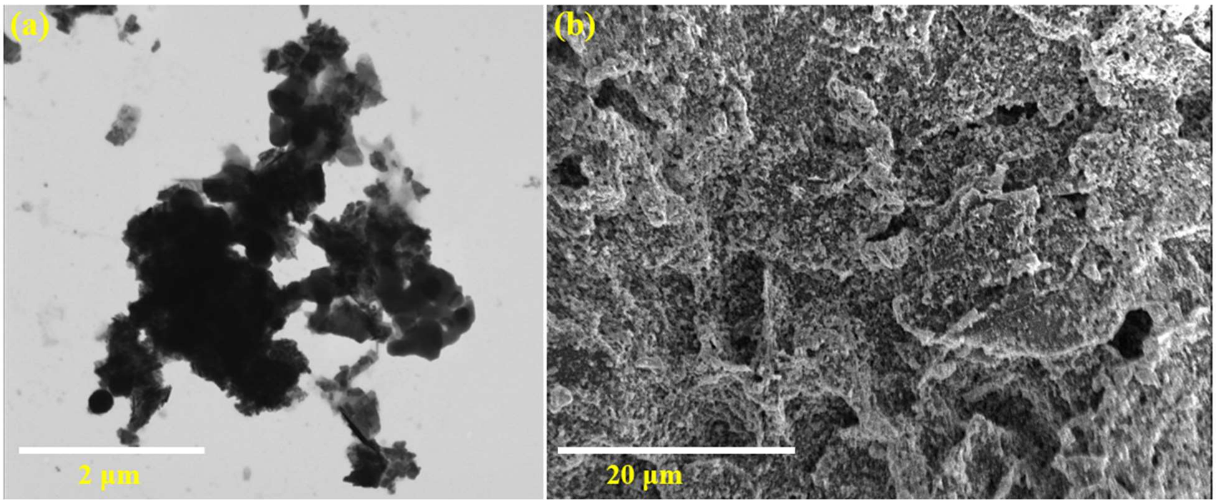

Figure S14: (a) Scanning transmission electron microscopic (STEM) images of Mo_2S_3-WS_2 after stability and (b) SEM images after stability for 30 h.

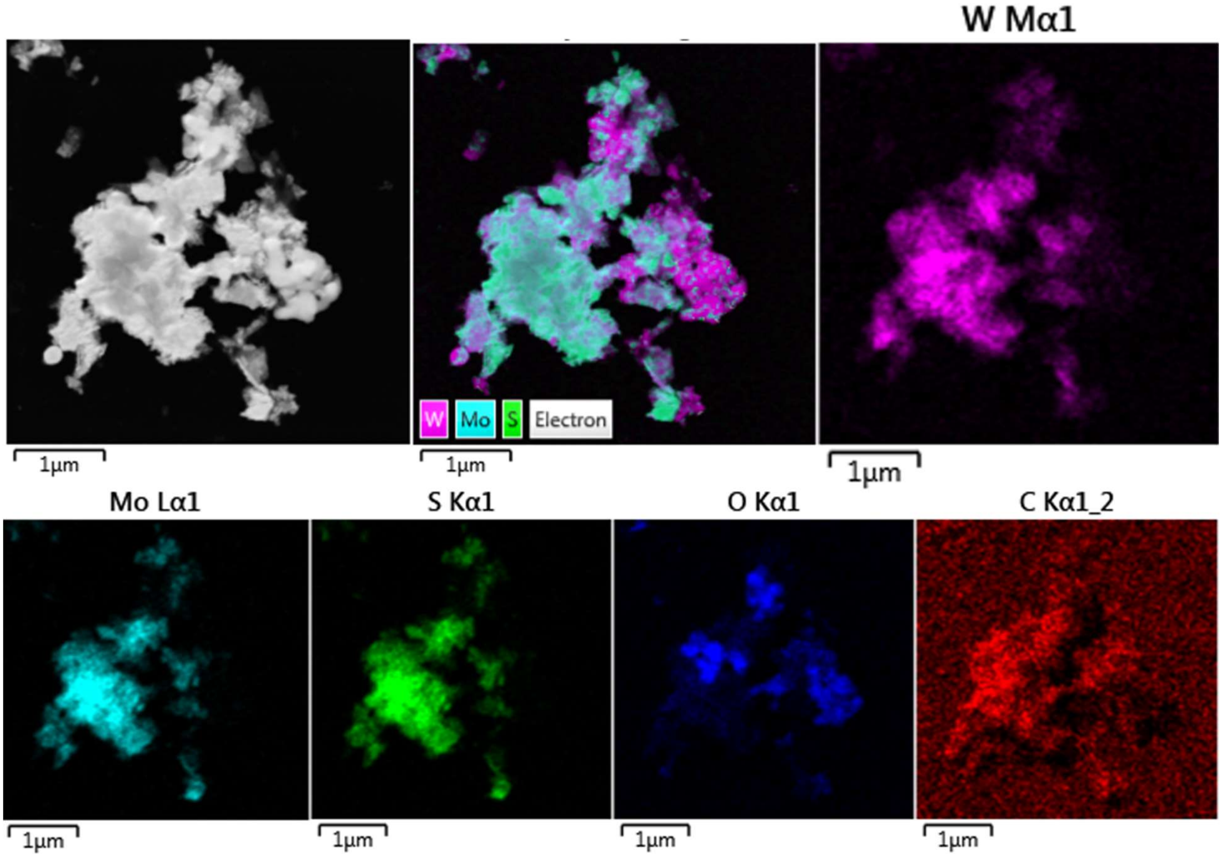

Figure S15: STEM-EDX mapping images of Mo_2S_3-WS_2 after 30 h of stability test.

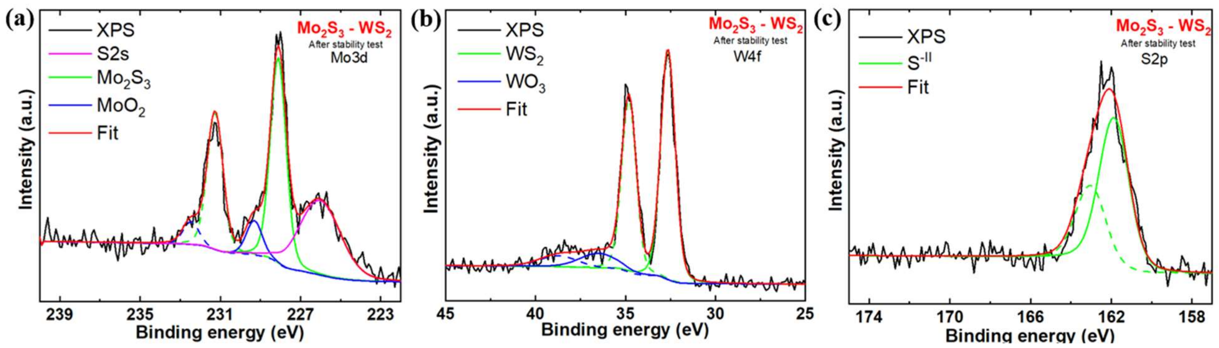

Figure S16: XPS high-resolution spectra of (a) Mo 3d, (b) W 4f and (c) S 2p of Mo_2S_3-WS_2 nanostructures after stability measurement for 30 h.

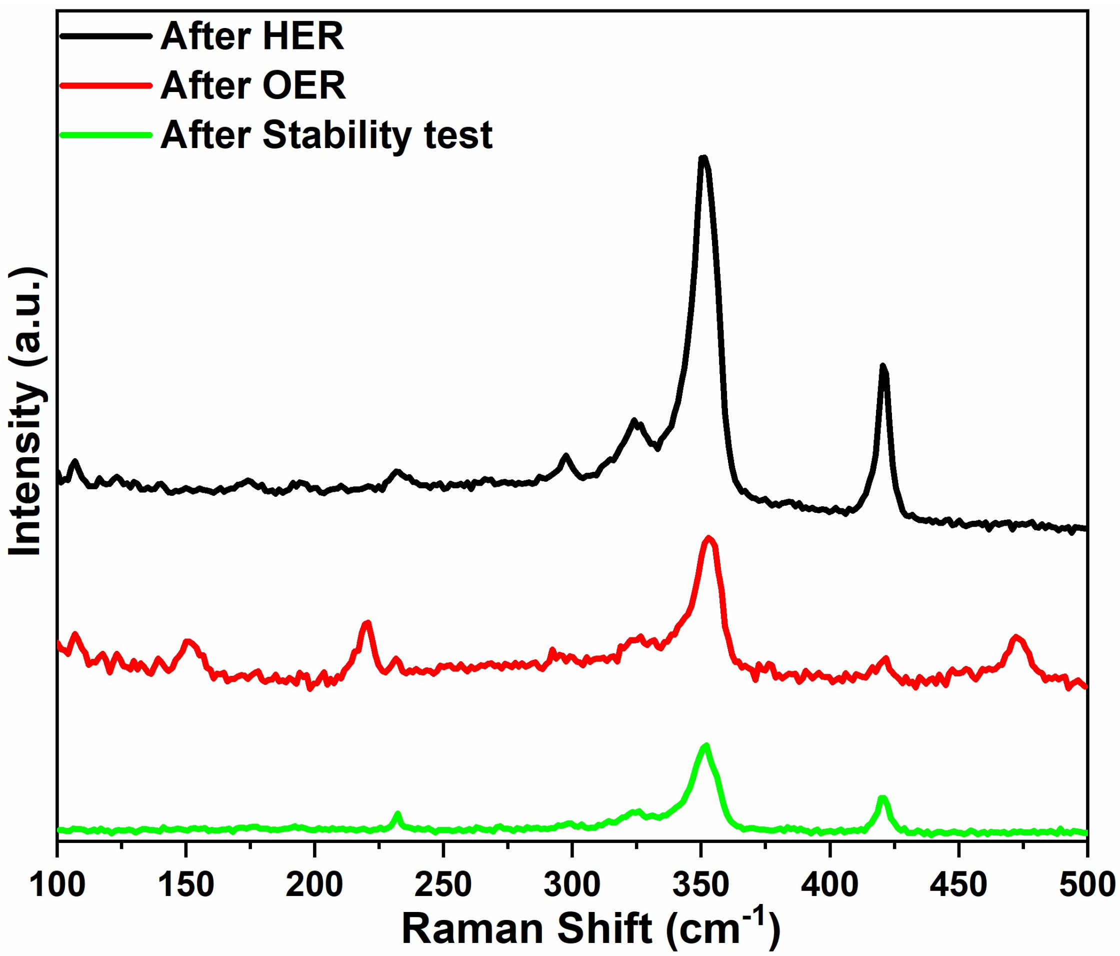

Figure S17: Raman spectroscopic images of Mo_2S_3-WS_2 nanostructures after HER, OER after 30 h of stability measurement.

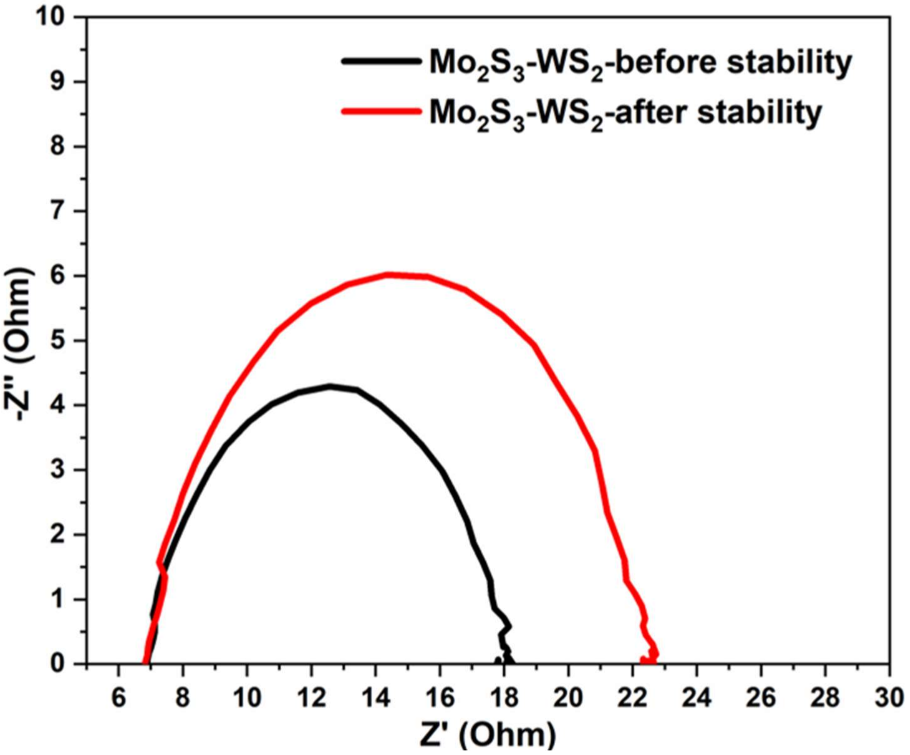

Figure S18: The electrochemical impedance spectra (EIS) of Mo_2S_3-WS_2 before and after 30 h of stability measurement.

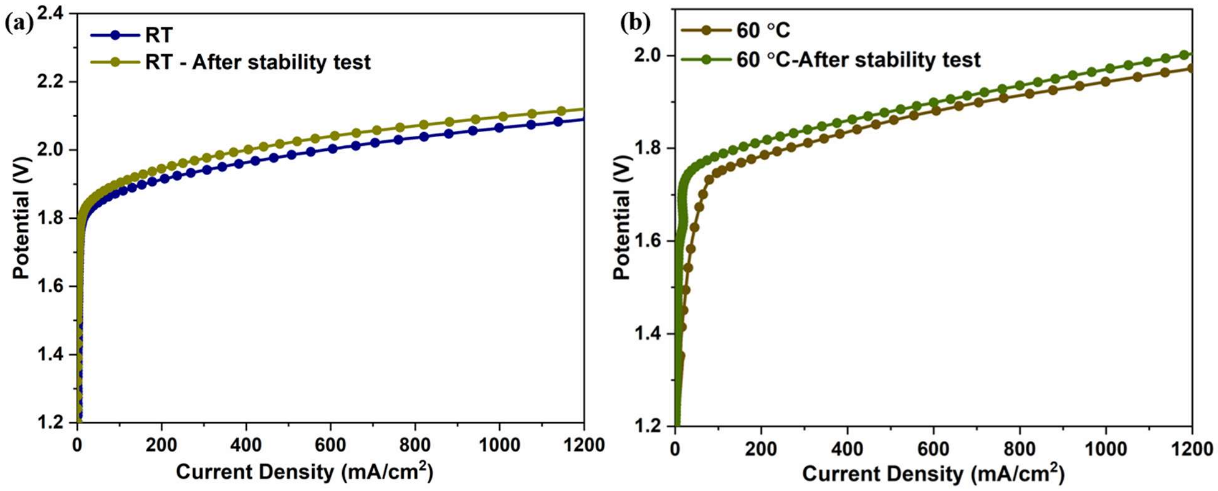

Figure S19: Polarization curves of a zero-gap alkaline water electrolyzer (Mo_2S_3-WS_2/NF || Mo_2S_3-WS_2/NF) in 6 M KOH at (a) room temperature and (b) 60 °C before and after the stability test.

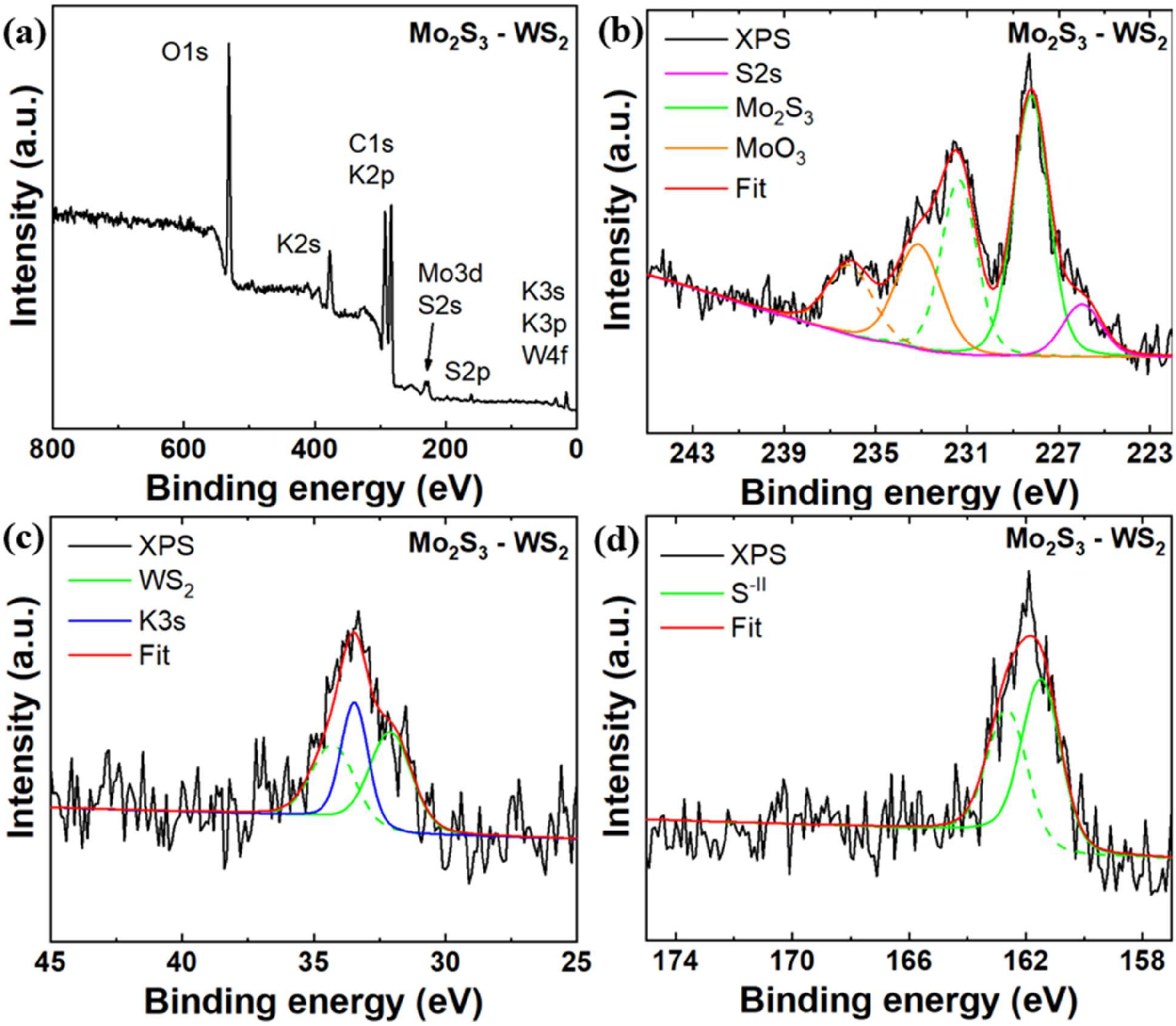

Figure S20: XPS (a) survey spectra of Mo_2S_3-WS_2 nanostructures and high-resolution spectra of (b) Mo 3d, (c) W 4f and (d) S 2p of Mo_2S_3-WS_2 nanostructures after performing stability test of in zero-gap alkaline water electrolyzer under continuous operation at 0.5 mA/cm^2 in 6 M KOH at RT for over 500 h.

References

1. H. Zhao, M. Liu, X. Du and X. Zhang, Synthesis of M-NiS/Mo_2S_3 (M=Co, Fe, Ce and Bi) nanoarrays as efficient electrocatalytic hydrogen evolution reaction catalyst in fresh and seawater, *Int. J. Hydrogen Energy*, 2024, **62**, 532-540, DOI: https://doi.org/10.1016/j.ijhydene.2024.03.077.

2. J. Li, M. Li, Y. Li, X. Guo and Z. Jin, Lotus-leaf-like $Bi_2O_2CO_3$ nanosheet combined with Mo_2S_3 for higher photocatalytic hydrogen evolution, *Sep. Purif. Technol.*, 2022, **288**, 120588, DOI: https://doi.org/10.1016/j.seppur.2022.120588.
3. L. Bo, L. Pu, Y. Hu, F. Nian, Z. Zhang, P. Li and J. Tong, Hydrangea like composite catalysts of ultrathin Mo_2S_3 nanosheets assembled on N, S-dual-doped graphitic biocarbon spheres with highly electrocatalytic activity for HER, *Int. J. Hydrogen Energy*, 2022, **47**, 6700-6709, DOI: https://doi.org/10.1016/j.ijhydene.2021.12.042.
4. B. Yang, F. Jin, X. Pan, X. Jin and Z. Jin, Directional Electron Transfer in $CuInS_2/Mo_2S_3$ S-Scheme Heterojunctions for Efficient Photocatalytic Hydrogen Production, *ACS Appl. Mater. Interfaces*, 2024, **16**, 36333-36342, DOI: https://doi.org/10.1021/acsami.4c05199.
5. X. Zhang, J. Zhang, X. Zha, Y. Luo, Y. Hu, G. Chen and X. He, Interfacial chemical bond and oxygen vacancies modulated Mo_2S_3/BiOBr high-low junctions for enhanced photocatalysis gatifloxacin degradation, *Appl. Surf. Sci.*, 2023, **641**, 158548, DOI: https://doi.org/10.1016/j.apsusc.2023.158548.
6. N. Qin, F. Dai, Y. Xue, D. Gao, Y. Liu, Y. Zhang, J. Chen and Q. Yang, Acanthosphere-like bimetallic sulfide Cu_9S_5/Mo_2S_3/NF as bifunctional catalyst for water splitting, *J. Electroanal. Chem.*, 2024, **964**, 118338, DOI: https://doi.org/10.1016/j.jelechem.2024.118338.
7. Z. L. Choong, B. T. Goh, M. L. Ooi, K. C. Lau, R. C. S. Wong and K. W. Tan, Tailoring the morphology of Mo_2S_3/MoP_2 composite thin films via aerosol assisted chemical vapor deposition for enhanced hydrogen evolution reaction performance, *Thin Solid Films*, 2024, **788**, 140150, DOI: https://doi.org/10.1016/j.tsf.2023.140150.
8. W. Liu, Y. Xiong, Q. Liu, X. Chang and J. Tian, The construction of S-scheme heterostructure in ultrathin $WS_2/Zn_3In_2S_6$ nanosheets for enhanced photocatalytic hydrogen evolution, *J. Colloid. Interface Sci.*, 2023, **651**, 633-644, DOI: https://doi.org/10.1016/j.jcis.2023.07.200.
9. Z. Ma, X. Ma, L. Zhang, H. Cheng and F.-N. Shi, 1T-WS_2/NCN photocatalyst with unique trion behavior and cyano-defects: Photocatalytic hydrogen evolution and mechanistic insights, *Sep. Purif. Technol.*, 2023, **325**, 124743, DOI: https://doi.org/10.1016/j.seppur.2023.124743.

10. K. Wang, N. Zhao, H. Xie, J. Wang, W. Xu and Z. Jin, Cocatalyst 1T-WS_2 assisted Prussian blue derivatives Ni-CdS to enhance photocatalytic hydrogen production driven by visible light, *Int. J. Hydrogen Energy*, 2024, **61**, 296-306, DOI: https://doi.org/10.1016/j.ijhydene.2024.02.265.
11. F. Liu, D. Zeng, Y. Tian, Y. Hu, T. Shen, Y. Gao and R. Guan, Ternary heterostructure Cu-$ZnIn_2S_4$/WO_3/WS_2 flower-like microspheres for highly-efficient photocatalytic hydrogen evolution under visible-light irradiation, *Appl. Surf. Sci.*, 2024, **642**, 158572, DOI: https://doi.org/10.1016/j.apsusc.2023.158572.
12. B. Wang, Y. Sun, X. Fan, W. Chen, X. Liu, L. Gao and L. Mao, In situ growth of 1T-WS_2 on ultrathin $Ti_3C_2T_x$ as a hybrid cocatalyst for enhancing the photocatalytic activity of CdS, *Appl. Surf. Sci.*, 2023, **615**, 156305, DOI: https://doi.org/10.1016/j.apsusc.2022.156305.
13. Y. Sun, B. Wang, X. Liu, L. Gao and W. Shangguan, Synthesis of Ternary Cross-Linked MoS_2/WS_2/CdS Photocatalysts for Photocatalytic H_2 Production, *Catalysts*, 2023, **13**, 1149, DOI: https://doi.org/10.3390/catal13081149.
14. T. Li, X. Wang, Z. Jin and N. Tsubaki, Tailoring Advanced CdS Anisotropy-Driven Charge Spatial Vectorial Separation and Migration via In Situ Dual Co-Catalyst Synergistic Layout, *Small*, 2024, **20**, 2311441, DOI: https://doi.org/10.1002/smll.202311441.
